# A porosity model for medical image segmentation of vessels

**DOI:** 10.1002/cnm.3580

**Published:** 2022-02-24

**Authors:** Vahid Goodarzi Ardakani, Alberto M. Gambaruto, Goncalo Silva, Ricardo Pereira

**Affiliations:** ^1^ Department of Mechanical Engineering University of Bristol Bristol UK; ^2^ IDMEC, Mechatronics Department University of Évora Évora Portugal; ^3^ Department of Neurosurgery Coimbra University Hospital Center, Faculty of Medicine, University of Coimbra Coimbra Portugal

**Keywords:** aortic arch, cerebral aneurysm, computational fluid dynamics, medical image segmentation, nasal cavity, porous medium, velocity thresholding, viscous resistance

## Abstract

A physics‐based medical image segmentation method is developed. Specifically, the image greyscale intensity is used to infer the voxel partial volumes and subsequently formulate a porous medium analogy. The method involves first translating the medical image volumetric data into a three‐dimensional computational domain of a porous material. A velocity field is then obtained from numerical simulations of incompressible fluid flow in the porous material, and finally a velocity iso‐surface provides the surface description of the target object. The approach is tested on CT images of eight patient‐specific cases, where cerebral aneurysms, nasal cavities (NC), and an aortic arch (AA) are the objects of interest. In the aneurysm cases, the results are compared against constant greyscale thresholding and manual segmentation. The manual segmentations of the aneurysms are validated by a clinical practitioner. Only a qualitative comparison is available for the NC, and the AA geometries. The results show that the proposed method is effective and capable of extracting the target object in a noisy domain. A sensitivity study is carried out to verify the method's performance with respect to modelling or user choices. The segmentation by the proposed method is also evaluated by performing computational fluid dynamics simulation, including a near‐wall flow analysis, to ensure that the segmented geometry and the resulting computed solution are representative and meaningful.

## Graphical abstract

A novel image segmentation method is proposed, in which the medical image intensity is translated into a porous model for subsequent computational fluid dynamics simulations. The desired object segmentation is then given as a fluid velocity magnitude iso‐surface. The segmentation is overall robust to low resolution and noisy image data, it can be readily generalised to different applications, and the method requires little user intervention.

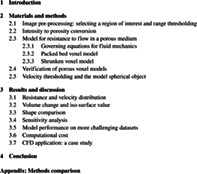

## 1 INTRODUCTION

Medical images play a crucial role in diagnosis, evaluation and treatment planning. Consequently, processing medical data has drawn much attention among researchers in terms of identifying and analysing desired features. For instance, image segmentation, as the delineation and extraction of distinct regions or objects, is one of the most demanding tasks in medical image analysis.

The accuracy of a segmentation is adversely affected by uncertainties present in the medical images. Images are prone to low resolution and partial volume effects, noise, intensity inhomogeneity introduced by image acquisition devices, as well as imaging artefacts. This makes it challenging and cumbersome to determine whether a segmentation is accurate, and how any error propagates in subsequent analysis. This concern arises often in some applications related to the cardiovascular or respiratory systems for example, where the near‐wall flow field is of great significance due to signalling pathways and mass transport. Hence, if the object surface is imprecisely obtained, the physiological and clinical implications could be significantly altered.^1^, ^2^, ^3^, ^4^, ^5^

The spectrum of available segmentation methods is quite broad, including manual and several automatic techniques. In manual segmentation, a user delineates objects of interest within the medical images, based on clinical knowledge and experience. This approach is time‐consuming and more critically it is not repeatable,^6^, ^7^, ^8^ however one can expect a high precision of the segmentation with high confidence. Manual segmentation by an experienced operator can typically provide the best possible segmentation, despite uncertainties present. The dramatic increase in medical data available and the demand for extracting complex features and organs, has led the research community to develop semi‐automated and automatic segmentation methods that not only are able to overcome some limitations of manual segmentation, but also speed up the image processing and image understanding tasks.^9^ These approaches can be classified generally by the underlying algorithm and model adopted, and can include threshold‐based methods, clustering techniques, deformable models, or machine learning and atlas based approaches.^9^, ^10^, ^11^, ^12^, ^13^, ^14^ In recent years image processing has been dominated by artificial neural network (ANN) methods, such as convolutional neural networks (CNN) architectures.^15^, ^16^, ^17^, ^18^ These methods however require large training datasets, which is often unfeasible, but importantly these are algorithm‐based methods and their success can vary, especially when generalisations are required.

Medical image acquisition and use, including their segmentation, is now a determining factor in different medical applications, however the images vary immensely from an analysis standpoint. Consequently, despite the progress made in this field, there is no universal image processing algorithm to meet the demands of various applications.^12^ This has spurred the development of segmentation tools which employ different algorithms and methods in order to provide versatility for users, making them predominantly rule or algorithm‐based^19^, ^20^, ^21^, ^22^, ^23^ rather than based on physical principles.^24^, ^25^

There is indeed a wide range of algorithm‐based image analysis and segmentation techniques. Conventional segmentation approaches such as level‐set and fast marching methods are based on partial differential equations whose solution specifies the position of a wave front propagating through the target object.^24^, ^26^ Region growing methods work based on the similarity of neighbouring pixels in the image, where the pixels with similar intensity are clustered. The growing process can be initialised with a user‐defined seed point. Watershed segmentation is a capable region‐based technique in which the image is treated like a topographic map, where the brightness of each pixel represents its elevation.^27^, ^28^ Edge or boundary‐based methods are also popular. Edge detectors locate and classify sharp discontinuities in the image where there are immediate changes in pixels' intensity or concentration. This process is usually performed by edge detection operators constructed like differential convolution kernels to be sensitive to large gradients in the image.^26^ Active contours (or snakes) are subsets of deformable models and are common in image analysis and are formulated as an energy minimising problem. First, an active and deformable contour whose energy depends on its shape and location within the image is defined. The active contour then evolves toward the target object edge by minimising the energy.^29^, ^30^

In this paper the medical image segmentation is motivated by a model of a physical process, namely flow in a porous medium. Specifically, we translate the image greyscale intensity variations due to partial volume effects as a domain partially occupied by fluid and by solid material, modelling this as a porous material. With this analogy, the image dataset is converted to a computational domain, and computational fluid dynamics (CFD) simulations of flow through the porous medium provides a velocity field. By subsequently selecting a velocity magnitude iso‐surface, the surface description of the object of interest can then be obtained.

To the best of our knowledge, a similar concept which leads to consistently accurate segmentations without manual intervention has not been adopted. The closest work is the voxel‐based CFD modelling and Lattice Boltzmann Methods (LBM) performed on upper and lower parts of human airways,^31^, ^32^, ^33^ however the focus was not image segmentation. As our test cases, we will first consider five patient‐specific datasets in which cerebral aneurysms (CA) are the target objects, and show that the developed method provides high precision, while being relatively automatic and robust. The model is then used to segment more anatomically complex shapes, specifically an aortic arch (AA) and two NC.

The paper is organised as follows. In Section 2 the overall methodology is detailed. Section 2.1 explains the medical image pre‐processing tasks, which involve identifying the region of interest and an approximate viable image intensity range. The translation from image intensity to porosity is presented in Section 2.2. The governing equations and the derivation of the semi‐empirical equation for resistance in the porous medium, as well as the CFD modelling of the porous domain, are outlined in Section 2.3. In Section 2.4 the *packed bed* and *shrunken voxel* porous resistance models are compared, indicating that and the *shrunken voxel* model provides a unique solution and is a viable porosity model. In Section 2.5 a thresholding value indicator is introduced to make the segmentation relatively automatic, and is based on a simple spherical object model. The results of the velocity iso‐surface thresholding are discussed in Section 3, and finally the concluding remarks are drawn in Section 4.

## 2 MATERIALS AND METHODS

Volumetric medical imaging data is typically formed by stacking a series of two‐dimensional images, which have been acquired with a slice thickness and spacing, or reconstructed from rotational projections. The slice thickness and spacing are chosen to ensure there is some overlap in the domain scanned. The three‐dimensional dataset provides a spatial sampling of the domain into individual voxels, which dimensions depend on the scanning sequence. The image intensity (which is commonly a scalar greyscale value) is constant for each voxel and represents volume averaged information. This averaging and the limited resolution available from the scanner can hamper medical image data analysis, and is commonly referred to as the partial volume effect.^34^

The image intensity for each voxel provides information of the averaged material properties contained in the volume of each voxel as well as due to the slice overlap. Taking as example a blood vessel, at the lumen‐tissue interface the delineation of the vessel boundary is challenging due to the partial volume effect, which results in a tessellation, and smearing of the image intensity. Indeed, at the interface regions the voxel intensity is a result of an average partial volume of fluid (VoF) and partial volume of tissue within the same voxel volume. We will model this partial volume as a porous medium.

Each voxel intensity is translated to a porous medium model; where there is a solid part associated with tissue as well as voids through which fluids may flow (for example air when considering medical images of the airways or blood in cardiovascular applications). If the voxel contains mostly tissue, then the porosity tends to zero, and conversely if the voxel contains mostly fluid then the porosity tends to unity. Flow through a porous medium is commonly modelled as flow across a granular *packed bed* of spherical particles, however this model is not suitable to represent a voxel partial volume, for which we can imagine the solid and fluid to occupy opposite regions of the voxel. We therefore develop a new porous model, discussed in Section 2.3.3, and termed *shrunken voxel* model (see Figure 4).

**FIGURE 1:**
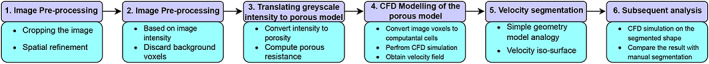
Flowchart of the steps followed for carrying out the velocity iso‐surface segmentation, based on translating the medical image greyscale dataset to a porous material model

We use this analogy to introduce a new method for medical image segmentation. Employing the shrunken voxel model for flow in a porous medium, we convert the medical image intensity to parameters of porous viscous resistance. Performing CFD we obtain a velocity field. The iso‐surface of velocity magnitude is then used as a tool for extracting the desired object surface definition. The main steps of the proposed medical image segmentation method are presented in Figure 1, and are summarised as follows:

1. *Image pre‐processing*: The original image dataset is cropped to the region of interest such that the target object lies inside. Depending on the image (the target object shape and size, and the image quality), the cropped region is upsampled using linear interpolation.
2. *Coarse thresholding*: To reduce severe noise and the size of the computational domain, a coarse image intensity thresholding operation is performed on the cropped region using a wide threshold range.
3. *Translating greyscale intensity to a porous model*: The porosity for each voxel is found through a simple linear relation between medical image intensity and the porosity. The porous model resistance is computed using a novel semi‐empirical equation, termed the *shrunken voxel* model.
4. *CFD modelling of the porous medium*: The voxels are now interpreted as computational cells in a finite volume solver and CFD simulations can be carried out. The cells with porosity less than 0.1 are considered effectively as solid for numerical reasons, and are excluded from the domain.
5. *Velocity segmentation*: An iso‐surface of velocity magnitude is used to define the shape of the target object. The choice of the iso‐value is guided by a simplified geometry model analogy.
6. *Use in subsequent analysis*: The virtual model obtained from the velocity magnitude iso‐surface segmentation can be used for subsequent studies.

To evaluate the success of the proposed method, several numerical tests on different patient specific geometries were carried out. The segmentations of the aneurysm cases were compared to manual segmentation by an experienced user and validated by a clinical expert. Additionally, to assess the propagation of segmentation uncertainty, computational haemodynamic simulations were carried out on a cerebral aneurysm and compared to previous work. As a critical comparison we focus on the near‐wall flow, which is sensitive to the no‐slip boundary definition.

### 2.1 Image pre‐processing: Selecting a region of interest and range thresholding

The medical image datasets evaluated in the present study are CT scans and consist of: five CA,^35^ two NC,^36^ one AA.^36^ Details of the image datasets are provided in Table 1. The cerebral aneurysm cases are used to explain and verify the proposed segmentation method by example, while segmentation results of the remaining datasets are used to show the method is versatile and performs generally well without further modification. The image pre‐processing stage involves two steps, which are now discussed.

**TABLE 1:** Image information for the dataset considered: 5 cerebral aneurysms (CA), 2 nasal cavities (NC), 1 aortic arch (AA). ${\bar{I}}_{\mathrm{ob}},$ ${\bar{I}}_{\mathrm{bg}},$ *σ*_*ob*_, and *σ*_*bg*_ are respectively the average intensity of the object or background, and standard deviation of the object or background

	Voxel size (mm)	Slice thickness (mm)	CNR, $\left(\frac{{\bar{I}}_{\mathrm{ob}}-{\bar{I}}_{\mathrm{bg}}}{\sigma_{\mathrm{bg}}}\right)$	SNR, $\left(\frac{{\bar{I}}_{\mathrm{ob}}-{\bar{I}}_{\mathrm{bg}}}{\sqrt{\frac{\sigma_{\mathrm{ob}}^{2}-\sigma_{\mathrm{ob}}^{2}}{2}}}\right)$
CA 1	0.5 × 0.5 × 0.4	0.625	46.62	12.02
CA 2	0.4 × 0.4 × 0.5	1	20.35	51.34
CA 3	0.363 × 0.363 × 0.4	0.625	26.87	87
CA 4	0.4 × 0.4 × 0.8	0.8	57.46	6.97
CA 5	0.443 × 0.443 × 0.5	1	60.01	30.49
NC 1	0.505 × 0.505 × 0.5	2	62.83	105.79
NC 2	0.473 × 0.473 × 0.55	0.75	30.34	43.79
AA 1	0.488 × 0.488 × 0.625	1	5.85	15.03

The first step in the pre‐processing is to crop the image to the region of interest, principally to reduce subsequent computational cost. The samples are then refined to have (0.1)^3^ mm voxel size using linear interpolation, in the case of the aneurysm datasets. This was effected primarily to allow for higher resolution numerical solutions of flow in porous media. This step can be ignored if the image voxel is fine enough.

In the second step an approximate range of viable image intensity is chosen, based on the object and background intensities. This is carried out in order to identify the regions correctly, such that we are segmenting the desired object within the image. The threshold range is chosen to be conservative to ensure the true geometry is retained, and there is no need for the user to select the limits carefully. A sensitivity study is carried out to verify this, and the ranges chosen for the test cases studied are presented in Table 2. Voxels with intensity outside the coarse thresholding range are discarded, further reducing the computational cost.

**TABLE 2:** Threshold values used in the velocity thresholding and image greyscale thresholding segmentation method. The inlet Reynolds number of each case is also presented. The image grayscale range for (cerebral aneurysm) CA 1–5, (nasal cavity) NC 1–2, and aortic arch (AA) 1 are: (−3024,3071), (−1024,2013), (−3024,3071), (0,622), (−1024,2745), (−1024,2976), (−1024,1989), (−3023,3071) respectively. Note that no plausible greyscale iso‐surface value exists for NCs and AA, as the image data is of poor quality and the object is too complex

	Re	Velocity thresholding			Greyscale thresholding
		Coarse threshold limits	Iso‐surface value, |u|(m/s)		Iso‐surface value, I
CA 1	0.059	(50 ≤ *I* ≤ 200)	10^−10^		100
CA 2	0.056	(90 ≤ *I* ≤ 200)	10^−10^		150
CA 3	0.089	(80 ≤ *I* ≤ 200)	10^−10^		150
CA 4	0.066	(100 ≤ *I* ≤ 200)	10^−10^		170
CA 5	0.132	(50 ≤ *I* ≤ 200)	10^−8^		160
NC 1	0.063	(−1000 ≤ *I* ≤ −200)	5 × 10^−6^		—
NC 2	0.045	(−1000 ≤ *I* ≤ −200)	5 × 10^−6^		—
AA 1	0.656	(−1200 ≤ *I* ≤ −1100)	10^−5^		—

**FIGURE 2:**
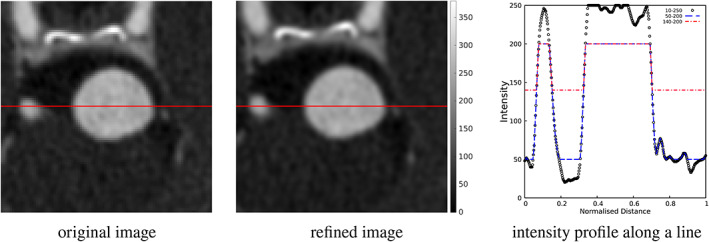
Comparison of intensity between the original (not upsampled) and refined (with upsampling) image, along with plot of intensity on an axial slice through the aneurysm, for patient CA 5. The image intensity is plotted along the red line marked. The refined image is upsampled to (0.1)^3^ mm using linear interpolation

As an example of the procedure, in Figure 2 the original and refined image for CA 5 are shown. Additionally, the intensity is plotted along a line crossing the aneurysm, together with two constant threshold demarcations for comparative purposes.

### 2.2 Intensity to porosity conversion

In computed tomography scans, the density of tissue has an approximately linear relation to the attenuation of the material.^37^, ^38^ Let us consider a voxel located on the interface between artery lumen and tissue, and in order to quantify how much of the voxel is occupied by its constituent components, we must first relate the image intensity of a voxel to the proportion of material constituents within it. The volume of a voxel can be written as the sum of the solid and fluid volume, or explicitly

(1) $$V_{v}=V_{s}+V_{f}$$

where *V*_v_, *V*_s_ and *V*_*f*_ are volume of the voxel, solid (tissue) and fluid (blood/air), respectively. The intensity can be defined by

(2) $$I_{v}=\frac{V_{s}I_{s}+V_{f}I_{f}}{V_{v}}=\frac{V_{s}}{V_{v}}I_{s}+\frac{V_{f}}{V_{v}}I_{f}$$

where *I*_v_, *I*_*s*_ and *I*_*f*_ are the intensity of voxel, solid and fluid, respectively. Introducing Equation 1 into Equation 2, we get

(3) $$I_{v}=\frac{\left(V_{v}-V_{f}\right)I_{s}+V_{f}I_{f}}{V_{v}}=\frac{V_{v}}{V_{v}}I_{s}-\frac{V_{f}}{V_{v}}I_{s}+\frac{V_{f}}{V_{v}}I_{f}=I_{s}+\frac{V_{f}}{V_{v}}\left(I_{f}-I_{s}\right)$$

*V*_*f*_/*V*_v_ is the ratio of the volume of the fluid over the volume of the voxel, which is the definition of porosity. In other words, the relation between voxel porosity and intensity can be written as

(4) $$\varepsilon =\frac{V_{f}}{V_{v}}=\frac{I_{v}-I_{s}}{I_{f}-I_{s}}$$

*I*_*f*_ and *I*_*s*_ can be considered the minimum and maximum intensity in the cropped image, regardless of whether we have a fluid‐tissue interface in the target object.

### 2.3 Model for resistance to flow in a porous medium

The pressure drop for fluid flow through porous media can be suitably described by Darcy's law, provided the Reynolds number is sufficiently low (*Re* ≪ 1). Darcy's law relates the pressure drop and fluid velocity as follows

(5) $$-\nabla p=\frac{\mu }{k_{p}}v_{s}$$

where *μ* is the fluid dynamic viscosity, and *k*_*p*_ is the permeability which is an intrinsic property of the porous medium. The *superficial velocity*, v_s_, is defined as the volume flow rate through a unit cross‐sectional area of the domain, comprising both solid and fluid regions. It has been shown that as the fluid velocity is increased, the relation between pressure drop and velocity becomes nonlinear.^39^ To describe this nonlinearity, Dupuit and Forchheimer^39^ included a quadratic term to generalise Equation 5, resulting in

(6) $$-\nabla p=\frac{\mu }{k_{p}}v_{s}+\mathrm{b\rho}v_{s}^{2}$$

Equation 6 is known as the Forchheimer equation. Factor *b* depends on the flow properties and medium of interest, and is determined experimentally from the Forchheimer graph.^39^

The semi‐empirical Ergun equation is an example of the Forchheimer equation, and is a well‐known relation for predicting pressure drop in granular packed beds of spherical particles. We will adopt this model to motivate and derive appropriate coefficients in Equation 6. The Ergun equation is a superposition of pressure drop due to *viscous* and *inertial* effects of the flow, which are known as Blake‐Kozeny and Burke‐Plummer equations, respectively.^40^, ^41^

**FIGURE 3:**
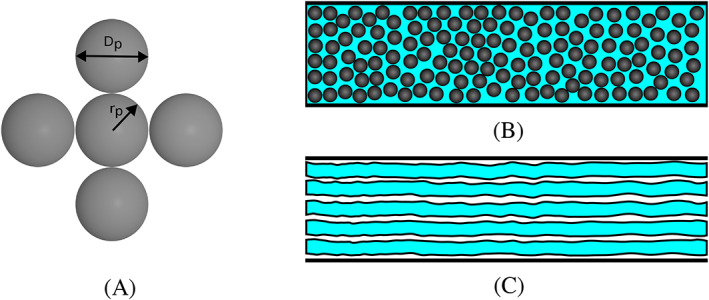
A packed bed of spherical particles (A), randomly distributed to make up a porous material (B), can be alternatively modelled as a tube bundle (C) when we consider a bulk flow direction along the axis of the tubes (here in horizontal direction)

Let us first consider the *viscous* forces. Flow through a granular packed bed can be considered as flow in a bundle of tangled cylindrical tubes assumed to have a uniform diameter along their length,^42^ as shown in Figure 3. The laminar flow in each cylinder is described by the Hagen‐Poiseuille equation for incompressible and Newtonian fluid. The Hagen‐Poiseuille equation is then generalised for the bundle of tubes by introducing the hydraulic radius, *R*_*h*_, and constant shape factor *k* to include the effect of arbitrary capillary cross‐section. The generalised Hagen‐Poiseuille equation is then written as

(7) $$v=\frac{\Delta {\mathrm{pR}}_{h}^{2}}{\mathrm{k\mu L}}$$

where *μ* is the fluid dynamic viscosity, *v* is termed the *physical velocity* and represents the average velocity of the flow within the tubes, and Δ*p* is the pressure drop along an equivalent channel of length *L*.^41^ The hydraulic radius can be expressed as the ratio of the available void space for the flow and the wetted surface as follows

(8) $$R_{h}=\frac{\text{cross}‐\text{sectional area normal to the flow}}{\text{wetted perimeter}}=\frac{\text{volume of fluid}}{\text{wetted surface}}$$

or equivalently

(9) $$R_{h}=\frac{V_{f}}{A_{s}}=\frac{\varepsilon V_{o}}{A_{s}}=\frac{\varepsilon V_{s}}{A_{s}\left(1-\varepsilon \right)}$$

where *V*_*f*_ is the VoF, *V*_s_ is the volume of solid, *V*_*o*_ is the volume of porous medium (i.e., *V*_*o*_ = *V*_*f*_ + V_s_), and *A*_*s*_ is the wetted surface. In this context, porosity is defined by

(10) $$\varepsilon =\frac{V_{f}}{V_{o}}$$

The ratio of the solid volume over the wetted surface is given by V_s_/*A*_*s*_ in Equation 9. For a single spherical particle making up the granular bed this ratio is given by

(11) $$\frac{V_{s}}{A_{s}}=\frac{\frac{4}{3}\pi r_{p}^{3}}{4\pi r_{p}^{2}}=\frac{r_{p}}{3}=\frac{D_{p}}{6}$$

where *D*_*p*_ is particle diameter (see Figure 3). Substituting this expression into Equation 9, the hydraulic radius for a granular porous media can be expressed as

(12) $$R_{h}=\frac{\varepsilon D_{p}}{6\left(1-\varepsilon \right)}$$

Substituting this expression for the hydraulic radius into Equation 7, the mean co‐axial velocity may be written as

(13) $$v=\frac{\varepsilon^{2}D_{p}^{2}}{36\mathrm{k\mu}{\left(1-\varepsilon \right)}^{2}}\;\frac{\Delta p}{L}$$

The physical velocity may be expressed as

(14) $$v=\frac{Q}{\varepsilon \;A_{\times }}=\frac{1}{\varepsilon }v_{s}$$

where *Q* is the volume flow rate and *A*_×_ is the cross‐sectional area of the domain. The ratio *Q*/*A*_×_ is the *superficial velocity*, v_s_. It is apparent from Equation 14 that in porous media the physical velocity is greater than the superficial velocity, since there is less space available for the fluid to flow. When superficial velocity is substituted into Equation 13, we obtain

(15) $$\frac{\Delta p}{L}=36k\frac{{\left(1-\varepsilon \right)}^{2}}{\varepsilon^{3}}\;\frac{\mu v_{s}}{D_{p}^{2}}=A\frac{{\left(1-\varepsilon \right)}^{2}}{\varepsilon^{3}}\;\frac{\mu v_{s}}{D_{p}^{2}}$$

where coefficient *A* is an empirical correction factor, which is proposed to be *A* = 150.^40^ The Blake‐Kozeny equation, which considers the viscous effects, is then given by

(16) $$\frac{\Delta P}{L}=150\frac{{\left(1-\varepsilon \right)}^{2}}{\varepsilon^{3}}\;\frac{\mu v_{s}}{D_{p}^{2}}$$

Let us now consider the *inertial* forces on the flow. It has been shown that the friction factor for highly turbulent internal flows no longer depends on Reynolds number.^42^ As such, Darcy's law cannot be attributed to the turbulent flow regime. In order to account for turbulent flow in the tubes, we start by expressing the volume flow rate for an incompressible and Newtonian fluid as

(17) $$Q=\frac{\pi D^{4}\Delta p}{128\mathrm{\mu L}}$$

where *D* is the tube diameter, *L* is the tube length, and Δ*p* is the pressure drop across the tube.^43^ Given that the volume flow rate is equal to velocity multiplied by cross‐sectional area (*Q* = *uA* = *uπD* ^2^/4), the pressure drop for flow in a pipe, in absence of body force, is given by

(18) $$\Delta p=32\mathrm{\mu v}\frac{L}{D^{2}}$$

In order to describe Equation 18 in terms of dimensionless quantities, one can divide both sides by the dynamic pressure, *ρv* ^2^/2, to obtain the dimensionless form as follows

(19) Δp12ρv2=32μLv/D212ρv2=64μρvDLD=64ReLD

This is often written as

(20) $$\Delta p=f\frac{L}{D}\frac{\rho v^{2}}{2}$$

where the dimensionless quantity

(21) $$f=\frac{\Delta p}{\frac{1}{2}\rho v^{2}}\frac{D}{L}$$

Is termed the friction factor.^43^ Using the hydraulic radius given by Equation 12, the friction factor can be rewritten as follows

(22) $$f=\frac{\Delta p}{\frac{1}{2}\rho v^{2}}\;\frac{R_{h}}{L}=\frac{\frac{\Delta P}{L}}{\frac{1}{2}\rho v^{2}}\;\frac{\varepsilon D_{p}}{6\left(1-\varepsilon \right)}=B\frac{\frac{\Delta P}{L}}{\rho v^{2}}\;\frac{\varepsilon D_{p}}{\left(1-\varepsilon \right)}$$

Introducing superficial velocity into Equation 22 and rearranging it for the pressure drop, we get

(23) $$\frac{\Delta p}{L}=B\frac{\left(1-\varepsilon \right)}{\varepsilon^{3}}\;\frac{\rho v_{s}^{2}}{D_{p}}$$

where coefficient *B* is an empirical correction factor, which is proposed to be *B* = 1.75,^40^ yielding

(24) $$\frac{\Delta p}{L}=1.75\frac{\left(1-\varepsilon \right)}{\varepsilon^{3}}\;\frac{\rho v_{s}^{2}}{D_{p}}$$

Equation 24 is known as the Burke‐Plummer equation, and takes into account the inertial effects.

The Ergun equation is obtained by addition of Blake‐Kozeny and Burke‐Plummer equations, hence the viscous and the inertial terms, and is given by

(25) $$\frac{\Delta p}{L}=\underset{\text{Viscous}\;\left(\text{Blake}‐\text{Kozeny}\right)}{\underbrace{A\frac{{\left(1-\varepsilon \right)}^{2}}{\varepsilon^{3}}\;\frac{\mu v_{s}}{D_{p}^{2}}}}+\underset{\text{Inertial}\;\left(\text{Burke}‐\text{Plummer}\right)}{\underbrace{B\frac{\left(1-\varepsilon \right)}{\varepsilon^{3}}\;\frac{\rho v_{s}^{2}}{D_{p}}}}$$

By re‐casting Equation 25 based on dimensionless numbers we obtain

(26) $$\frac{\frac{\Delta p}{L}}{\frac{\rho v_{s}^{2}}{D_{p}}\;\frac{\left(1-\varepsilon \right)}{\varepsilon^{3}}}=A\frac{\mu }{\frac{\rho v_{s}D_{p}}{1-\varepsilon }}+B$$

which can be expressed as

(27) fb=AReb+B

where *f*_*b*_ is the packed‐bed friction factor, and *Re*_*b*_ the packed bed Reynolds number is defined as

(28) Reb=ρvsDpμ11−ε

The constants *A* and *B* are found by plotting *f*_*b*_ against *Re*_*b*_ for experimental data.^42^, ^44^

#### 2.3.1 Governing equations for fluid mechanics

To model the fluid flow in a porous medium, one could solve the flow equations within the microscopic void structures; however this is impractical for large problems. A practical approach is to average the microscopic transport equations over a control volume whose size is much larger than the characteristic length of pore structures but much smaller than the problem domain, the so‐called representative elementary volume (REV).^45^ This homogenisation procedure is well documented in the literature,^45^, ^46^, ^47^, ^48^, ^49^, ^50^, ^51^ and we report only the main results to assist our presentation.

The equations for conservation of mass and linear momentum for a fluid flow are given by the continuity and Navier–Stokes equations, as

(29) $$\frac{\partial \rho }{\partial t}+\nabla \cdot \left(\rho u_{f}\right)=0$$

(30) $$\frac{\partial \left(\rho u_{f}\right)}{\partial t}+\nabla \cdot \left(\rho u_{f}u_{f}\right)=-\nabla p_{f}+\nabla \cdot \left(T_{f}\right)+F$$

where **u**_*f*_ is the microscopic fluid velocity, *p*_*f*_ is the pressure, **T**_**f**_ is the stress tensor, and **F** is the body force. By applying averaging theorems^45^, ^49^, ^52^ and adopting the finite volume approach, one can obtain the following equations for momentum and continuity equations

(31) $$\frac{\partial }{\partial t}\left({\;}_{V}\int \rho \varepsilon \right)\mathrm{dV}+\oint_{A}\rho v_{s}\cdot ds=0$$

(32) $$\frac{\partial }{\partial t}\left(\int_{V}\rho v_{s}\right)\mathrm{dV}+\oint_{A}\rho v_{s}⊗v_{s}\cdot ds=-\oint_{A}pI\cdot ds+\oint_{A}T\cdot ds+\int_{V}f_{b}\mathrm{dV}+\int_{V}f_{p}\mathrm{dV}$$

where *V* represents the control volume, **s** is the surface area vector, **f**_*b*_ encapsulates all the body forces acting on the fluid except the porous resistance, **I** is the identity matrix, and **f**_*p*_ is the porous medium resistance force.^53^ The equations may be further simplified by considering the fluid to be incompressible and Newtonian. The porous medium resistance force **f**_*p*_ is defined as

(33) $$f_{p}=-P\cdot v_{s}$$

where *P* is the porous medium resistance tensor consisting of two components

(34) $$P=P_{v}+P_{i}|v_{s}|$$

where **P**_*v*_ and **P**_*i*_ are viscous and inertial resistance tensors, respectively. Comparing Equations 33, 34, and 6, the viscous and inertial resistance for the Forchheimer equation can be written as

(35) $$P_{v}=\frac{\mu }{k_{p}}$$

(36) $$P_{i}=\mathrm{b\rho}v_{s}$$

The Ergun equation (Equation 25) is one example of Forchheimer equation (Equation 6) for a particular class of flow, that is packed beds^42^ where **P**_*v*_ and **P**_*i*_ are defined by

(37) $$P_{v}=150\frac{{\left(1-\varepsilon \right)}^{2}}{\varepsilon^{3}}\;\frac{\mu }{D_{p}^{2}}$$

(38) $$P_{i}=1.75\frac{\left(1-\varepsilon \right)}{\varepsilon^{3}}\;\frac{\rho v_{s}}{D_{p}}$$

When it comes to finding the similarity between a voxel and a granular porous medium, two approaches, namely a *packed bed voxel* or a *shrunken voxel* model, can be considered. In the packed bed voxel model, a voxel is assumed to contain granular spherical particles while in the shrunken voxel model, a voxel is divided into two opposing regions for the solid and fluid. In both models, the voxel is converted to a porous medium based on its intensity. The main challenge to obtain the velocity field is predicting the extra pressure drop, and consequently the resistance terms, arising from porosity, namely **P**_*v*_ and **P**_*i*_. This can be achieved by following the same approach used to derive the Ergun equation and finding the equivalent Forchheimer equation (Equation 6) for the two voxel‐based models.

#### 2.3.2 Packed bed voxel model

**FIGURE 4:**
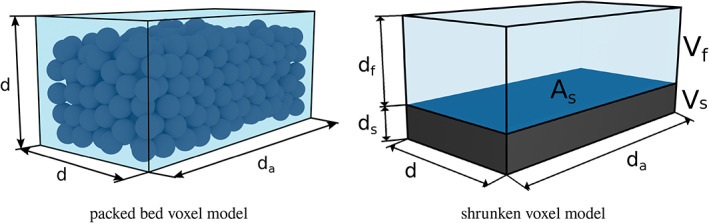
Schematic of a voxel with particles inside (left), and shrunken voxel with tissue (right). The notation is as follows: d) height and width of the voxel, d_*a*_) depth of the voxel, d_*f*_) height of the fluid region, d_*s*_) height of the solid region, V_*f*_) volume of the fluid region, V_s_) volume of the solid region, A_*s*_) wetted surface

In this approach, we assume the voxel to contain some spherical particles, as shown in Figure 4. The hydraulic radius is a key component in the Ergun equation, which needs to be specified according to the modelling assumptions. In the packed bed voxel model, the volume of tissue in the voxel equals the volume of spherical particles, hence

(39) Volume of the spherical particles=Volume of the solid part within the voxel

or mathematically

(40) $$N\frac{4}{3}\pi r_{p}^{3}=\left(1-\varepsilon \right)d^{2}d_{a}$$

which can be rearranged to yield

(41) $$r_{p}=0.62N^{-\frac{1}{3}}{\left(\left(1-\varepsilon \right)d^{2}d_{a}\right)}^{\frac{1}{3}}$$

where *r*_*p*_ is the radius of small particles in the voxel similar to the radius used in deriving the Ergun equation (see Figure 3), *d* is the length and width of the voxel (taken to be the same for simplicity, but is also the case here since the two‐dimensional image pixel dimensions is square), *d*_*a*_ is the depth of the voxel, and *N* is the number of particles in the voxel. Using Equation 11 and 41, the ratio V_s_/*A*_*s*_ can be obtained as

(42) $$\frac{V_{s}}{A_{s}}=\frac{r_{s}}{3}=\frac{0.62}{3}N^{-\frac{1}{3}}{\left(\left(1-\varepsilon \right)d^{2}\;d_{a}\right)}^{\frac{1}{3}}=0.206N^{-\frac{1}{3}}{\left(\left(1-\varepsilon \right)d^{2}\;d_{a}\right)}^{\frac{1}{3}}$$

Next, Equation 9 yields the hydraulic radius as follows

(43) $$R_{h}=\frac{V_{s}}{A_{s}}\frac{\varepsilon }{\left(1-\varepsilon \right)}=0.206N^{-\frac{1}{3}}{\left(\left(1-\varepsilon \right)d^{2}\;d_{a}\right)}^{\frac{1}{3}}\frac{\varepsilon }{1-\varepsilon }=0.206N^{-\frac{1}{3}}\frac{\varepsilon {\left(d^{2}\;d_{a}\right)}^{\frac{1}{3}}}{{\left(1-\varepsilon \right)}^{\frac{2}{3}}}$$

and consequently

(44) $$R_{h}^{2}={\left(0.206N^{-\frac{1}{3}}\right)}^{2}{\left(\frac{\varepsilon \left(d^{2}\;d_{a}\right)}{{\left(1-\varepsilon \right)}^{\frac{2}{3}}}\right)}^{2}={\left(0.206N^{-\frac{1}{3}}\right)}^{2}\frac{\varepsilon^{2}}{{\left(1-\varepsilon \right)}^{\frac{4}{3}}}{\left(d^{2}\;d_{a}\right)}^{\frac{2}{3}}$$

Substituting Equation 44 into Equation 7 and then merging the constants, for the pressure drop we find

(45) $$\frac{\Delta P}{L}=A\frac{{\left(1-\varepsilon \right)}^{\frac{4}{3}}}{\varepsilon^{2}}\frac{\mu \;v}{{\left(d^{2}\;d_{a}\right)}^{\frac{2}{3}}}$$

where *A* is a constant that needs to be determined. Using the hydraulic radius in Equation 43 and the friction factor equation (i.e., Equation 21), the inertial part of Ergun equation is defined by

(46) $$\frac{\Delta p}{L}=\frac{f\frac{1}{2}\rho v^{2}}{R_{h}}=\frac{f\frac{1}{2}\rho v^{2}}{0.206N^{-\frac{1}{3}}\frac{\varepsilon {\left(d^{2}d_{a}\right)}^{\frac{1}{3}}}{{\left(1-\varepsilon \right)}^{\frac{2}{3}}}}=\frac{\frac{1}{2}f}{0.206N^{-\frac{1}{3}}}\frac{{\left(1-\varepsilon \right)}^{\frac{2}{3}}}{\varepsilon }\frac{\rho v^{2}}{{\left(d^{2}d_{a}\right)}^{\frac{1}{3}}}=B\frac{{\left(1-\varepsilon \right)}^{\frac{2}{3}}}{\varepsilon }\frac{\rho v^{2}}{{\left(d^{2}d_{a}\right)}^{\frac{1}{3}}}$$

The complete equation for the pressure drop again is found by superposition of Equations 45 and 46 as follows

(47) $$\frac{\Delta P}{L}=A\frac{{\left(1-\varepsilon \right)}^{\frac{4}{3}}}{\varepsilon^{2}}\frac{\mathrm{\mu v}}{{\left(d^{2}d_{a}\right)}^{\frac{2}{3}}}+B\frac{{\left(1-\varepsilon \right)}^{\frac{2}{3}}}{\varepsilon }\frac{\rho v^{2}}{{\left(d^{2}d_{a}\right)}^{\frac{1}{3}}}$$

The friction factor, bed Reynolds number, and their relation are then respectively given by

(48) fb=ΔpLε2d2da13ρv21−ε23=AReb1−ε23+Bε

(49) Reb=ρvd2da13μ

#### 2.3.3 Shrunken voxel model

In this model, each voxel is considered to be partially occupied by the solid (tissue) while the rest is occupied by the fluid, as shown schematically in Figure 4. This model is appealing due to its similarity to the partial volume effect for medical image data in which voxels located on the boundary between two objects are partitioned in a similar fashion. In this model the hydraulic radius can be defined as

(50) $$R_{h}=\frac{V_{f}}{A_{s}}=\frac{\varepsilon d^{2}d_{a}}{{\mathrm{dd}}_{a}}=\mathrm{\varepsilon d}$$

Using this hydraulic radius in Equation 7, the viscous part of the Ergun equation for the shrunken voxel approach is given by

(51) $$\frac{\Delta p}{L}=A\frac{\mu }{\varepsilon^{2}d^{2}}v$$

The pressure drop for the inertial part is obtained by again using the hydraulic radius (Equation 50) in Equation 21 as

(52) $$\frac{\Delta p}{L}=B\frac{\rho }{\mathrm{\varepsilon d}}v^{2}$$

The pressure drop is obtained by the addition of Equation 51 and 52 as

(53) $$\frac{\Delta p}{L}=A\frac{\mu }{\varepsilon^{2}d^{2}}v+B\frac{\rho }{\mathrm{\varepsilon d}}v^{2}$$

Therefore, the friction factor and the bed Reynolds number are given by

(54) fb=ΔpLρv2εd=AεReb+B

(55) Reb=ερvdμ

### 2.4 Verification of porous voxel models

The Ergun equation is semi‐empirical since the coefficients appearing in the equations, namely *A* and *B*, require fitting to experimental data. The procedure to find constants of the Ergun Equation^40^ involves conducting an extensive number of experiments where the pressure drop is measured across packed columns for wide ranges of flow rate. In order to set the constants, the friction factor (*f*_*b*_) is plotted versus bed Reynolds number (*Re*_*b*_), and the constants are then determined using the least square fit approach. We follow the same steps to find the constants *A* and *B* in Equations 48 and 54, resorting to high resolution numerical solutions of flow in a single voxel as the test set‐up instead of running experiments on packed beds.

Let us consider a voxel located on the edge of two objects, like the shrunken voxel shown in Figure 4. Let us denote the height of an empty voxel by *d*, the height of the tissue region, hence the solid region by *d*_*s*_, and the height of the fluid region by *d*_*f*_, such that

(56) $$d=d_{f}+d_{s}$$

According to the definition of porosity (Equation 10), the height of the fluid and the solid region for a voxel can be expressed by

(57) $$d_{f}=\mathrm{\varepsilon d}$$

(58) $$d_{s}=\left(1-\varepsilon \right)d$$

To verify the relation between the friction factor and the bed Reynolds number, several numerical simulations were performed on a series of two‐dimensional voxel geometries with four main height diameters, *d* = 0.25, 0.5, 0.8, 1.0 mm. The inlet and outlet boundaries were set to be periodic, and a two‐dimensional fully developed half parabolic profile for velocity was prescribed at the inlet section. The bottom and the top boundaries of the voxel were considered to be no‐slip and symmetric, respectively. All the simulations were carried out for 15 inlet velocities between 0.0005 m/s and 4 m/s. The flow regime was considered to be laminar. A structured grid with a finer spatial resolution toward the no‐slip wall was generated for each geometry. The height of the first mesh layer was 10^−4^ mm, and the average cell area was 3.5 × 10^−5^ mm^2^. The finite volume solver STAR‐CCM+ 13.04.010‐r8 (Siemens) was used to perform the simulations. The effect of porosity on the pressure drop was captured by conducting 10 sets (varying porosity) of 15 simulations (varying inlet velocity) each for 4 different fluid region heights defined in Equation 57. The pressure drops obtained from simulations are used to compute the friction factor.

**FIGURE 5:**
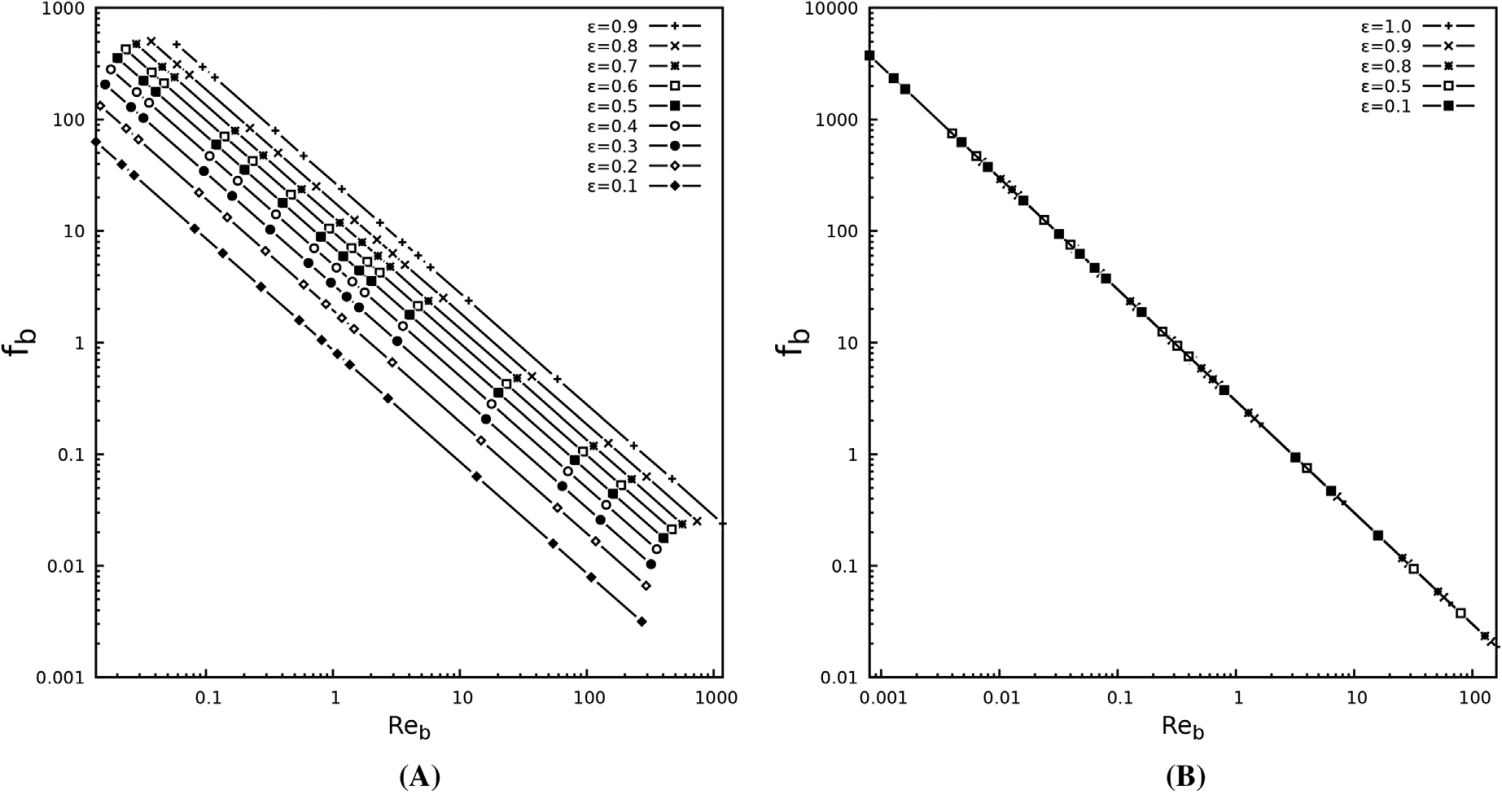
Friction factor vs bed Reynolds number for two approaches. (A) packed bed voxel approach; *f*_*b*_ and *Re*_*b*_ are described in Equations 48 and 49. (B) shrunken voxel approach; *f*_*b*_ and *Re*_*b*_ are mentioned in Equations 54, and 55

In Figure 5, the friction factor and bed Reynolds number for *d* = 0.25 mm are plotted. A comparison between Figure 5 and the result reported in^40^, ^42^, ^44^ reveals that the effect of inertial term is negligible, as the horizontal asymptote related to the Bruke‐Plummer equation does not appear in the plot.

Results for the packed bed voxel, using different porosities results in different relations between *f*_*b*_ and *Re*_*b*_, as seen by the different lines in Figure 5A. This implies that the packed bed voxel model is not suitable to correlate a general equation for estimating the pressure drop in the voxel subjected to partial volume effects. Conversely, Figure 5B shows that the results of the shrunken voxel model lie on the same line for different porosities. Performing a curve fit on the results in Figure 5B, the constants in Equation 53 are determined to be *A* = 3, while the constant *B* is negligible as was expected, hence *B* = 0.

Let us now consider the pressure drop in a voxel without solid (tissue) as a reference voxel, in which there is no pressure drop due to porosity. We can assume that the pressure drop caused by porosity in a porous voxel, partially filled with tissue, is equal to the difference between the pressure drop in the shrunken voxel and the reference voxel. Given the fact that *ε* = 1 for the reference (entirely filled by fluid) voxel, we can rewrite Equation 51 (viscous part) as follows

(59) $$\begin{array}{l} \frac{\Delta p}{L}=A\frac{\mu }{\varepsilon^{2}d^{2}}v-A\frac{\mu }{d^{2}}v\\ =A\frac{\left(1-\varepsilon^{2}\right)}{\varepsilon^{2}d^{2}}\mathrm{\mu v} \end{array}$$

Similarly, Equation 52 (inertial part) can be rewritten as

(60) $$\begin{array}{l} \frac{\Delta p}{L}=B\frac{\rho }{\mathrm{\varepsilon d}}v^{2}-B\frac{\rho }{d}v^{2}\\ =B\frac{\left(1-\varepsilon \right)}{\mathrm{\varepsilon d}}\rho v^{2} \end{array}$$

Adding Equations 59 and 60, the pressure drop caused by porosity in a porous voxel can be defined by

(61) $$\frac{\Delta p}{L}=A\frac{\left(1-\varepsilon^{2}\right)}{\varepsilon^{2}d^{2}}\mathrm{\mu v}+B\frac{\left(1-\varepsilon \right)}{\mathrm{\varepsilon d}}\rho v^{2}$$

Equation 61 applies for a single voxel. To generalise this estimate, the physical velocity is replaced by the superficial velocity, using Equation 14 (i.e., *v* = v_s_/*ε*) as follows

(62) $$\frac{\Delta p}{L}=A\frac{\left(1-\varepsilon^{2}\right)}{\varepsilon^{3}d^{2}}\mu v_{s}+B\frac{\left(1-\varepsilon \right)}{\varepsilon^{3}d}\rho v_{s}^{2}$$

As the constant for inertial resistance part in Figure 5 was negligible, we only consider viscous resistance term which is given by

(63) $$P_{v}=3\frac{\left(1-\varepsilon^{2}\right)}{\varepsilon^{3}d^{2}}\mu$$

This equation is then substituted into Equation 34 for subsequent simulations of fluid flow in a porous medium representation of the medical images.

Finally we remark that the equation for the porous viscous forces given by Equation 63 is singular for small porosity. Indeed, the viscous resistance tends to infinity when porosity tends to zero, which is equivalent of a voxel filled completely with tissue. Numerically it is difficult to consider values tending to infinity, and this requires setting a minimum limit for porosity, for which we would expect negligible flow within the voxel. For simplicity, we consider this limit to be 0.1, meaning a voxel with porosity *ε* ≤ 0.1 is assumed to be entirely solid with no flow inside. Such voxels are removed from the computational domain since they contain no fluid fraction. Conversely if *ε* = 1, the voxel is entirely occupied by fluid and there is no viscous resistance from the porosity term.

### 2.5 Velocity thresholding and the model spherical object

At this stage, the numerical simulation is performed and the velocity field inside the domain is obtained. The computational domain here is the converted medical image to the porous material model. In the medical images of computed tomography angiography (CTA), the boundary between vessels and the surrounding tissue is marked by a difference in the image greyscale intensity. This is translated to the porous resistance model, and hence we can expect the computed velocity to vary considerably across such boundaries, and it is our goal now to identify a velocity iso‐surface threshold which lies on this boundary. A common approach to identify an edge is to consider the spatial gradients of image intensity, as commonly adopted in PDE‐based anisotropic diffusion filters to specify the diffusion coefficients^54^ or the diffusion time.^55^, ^56^, ^57^

The numerical simulations of fluid flow in the porous medium analogy of the medical images will provide a high resolution velocity field, and we are now in a position to select a velocity magnitude iso‐surface which represents the object of interest. At this point several options would be available if we consider the computed velocity magnitude as equivalent scalar to an image greyscale intensity. Here, for simplicity, we look to select a velocity magnitude iso‐surface to define the object boundary. The proposed approach is to observe the changes in geometry (such as volume, surface area or diameter) as different velocity magnitude threshold values are chosen to extract the iso‐surface, and by observing these trends we may identify different behaviours.^55^ Let us explain the method through example, considering the CA as test cases, though it may be applied to any object in the image.

Having a model generally simplifies the analysis and in order to derive an automatic approach, we equate the volume and area of the segmented aneurysm to those of a sphere (as an idealised aneurysm) as follows

(64) $$\frac{4}{3}\pi {\left(R_{0}+\mathrm{dR}\right)}^{3}=V_{\text{aneurysm}}$$

(65) $$2\pi {\left(r_{0}+\mathrm{dr}\right)}^{2}=A_{\text{aneurysm}}$$

where the volume and area of the aneurysm from velocity thresholding are denoted by *V*_aneurysm_ and *A*_aneurysm_. Here *R*_0_ and *r*_0_ are initial radii of the model spherical aneurysm, whose volume and area are equal to those of the iso‐surface of |**u**| = 10^−5^ m/s (one order of magnitude less than the inlet velocity). For the initial iso‐surface value for some objects with complex shapes, such as the NC, it is better to use a velocity iso‐surface value of the inlet velocity to find the initial radii. *dR* and *dr* are respectively the changes in the radius of the model spherical aneurysm, when matching the volume and area of the velocity iso‐surface segmentation. These changes are obtained by varying the velocity iso‐surface values from |**u**| = 10^−14^ m/s to |**u**| = 10^−6^ m/s in Equations 64 and 65. One should note that in general *R*_0_ ≠ *r*_0_ and *dR*≠*dr* since these are found through equating either volume or area to the spherical model.

In practice we observe the trends of how *dR* and *dr* vary with decreasing velocity iso‐surface value, aiming to identify possible turning points and rapid changes in slope in the plot of *dr* and *dR* versus iso‐surface value. The reason for this is analogous to the adaptive filtering proposed in,^55^ namely as the iso‐surface value is gradually changed, the corresponding change in volume and surface area of the segmented object will vary at two rates due to: (i) the removal of noise, and (ii) the reduction of effective vessel diameter. Since noise in medical images occurs at a smaller spatial scale than the object, it will be responsible for higher gradients of volume and area change with varying *dR* and *dr*. Once the noise is effectively excluded, the further gradual changes observed are related to a reduction of effective vessel diameter. Identifying changes in the gradients, hence observing changes in a trend (i.e. second order derivatives and points of inflexion), will determine the iso‐value most appropriate for the segmentation.

## 3 RESULTS AND DISCUSSION

**FIGURE 6:**
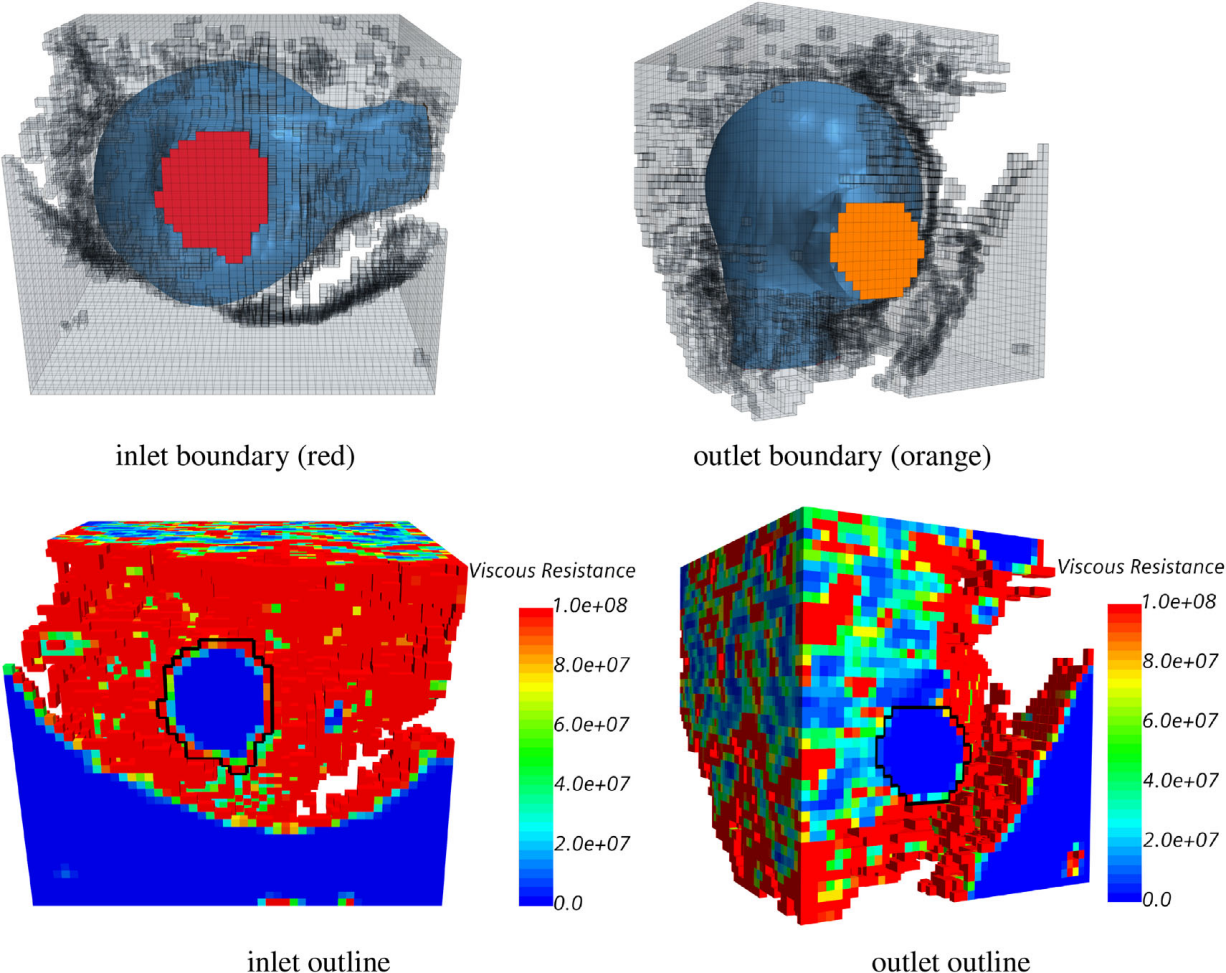
Computational domain for the original (prior to upsampling) image of CA 5. Top row: the blue surface is the result of the velocity segmentation, while the red and orange boundaries are the inflow and outflow sections, respectively. The transparent (grey) cells in this figure are part of the computational domain, which results from the initial user choice of coarse intensity thresholding. Bottom row: map of viscous resistance (*kg*/*m* ^3^ *s*) on the surface of the computational domain. The black closed contours delineate the inflow and outflow sections from the remaining no‐slip wall boundary

Numerical simulations for steady‐state flow in the porous medium model were carried out on a regular grid, which corresponds to the upsampled medical image voxels. A uniform and constant velocity magnitude of 10^−4^ m/s was imposed at the inlets, such that the Reynolds numbers at the inlet section are less than 1 and are detailed in Table 2. Low Reynolds number flow was chosen to ensure we would have creeping flow, with no flow separation nor secondary flow structures (e.g. vortices), allowing the flow to fill the domain based on the porosity and without influence of flow inertia. The outlet boundary condition was set to be zero pressure, and the walls were set to no‐slip boundaries. The inlet and outlet boundaries and their relation to the viscous resistance distribution on the wall for the original (not upsampled) data of CA 5 are presented in Figure 6.

### 3.1 Resistance and velocity distribution

**FIGURE 7:**
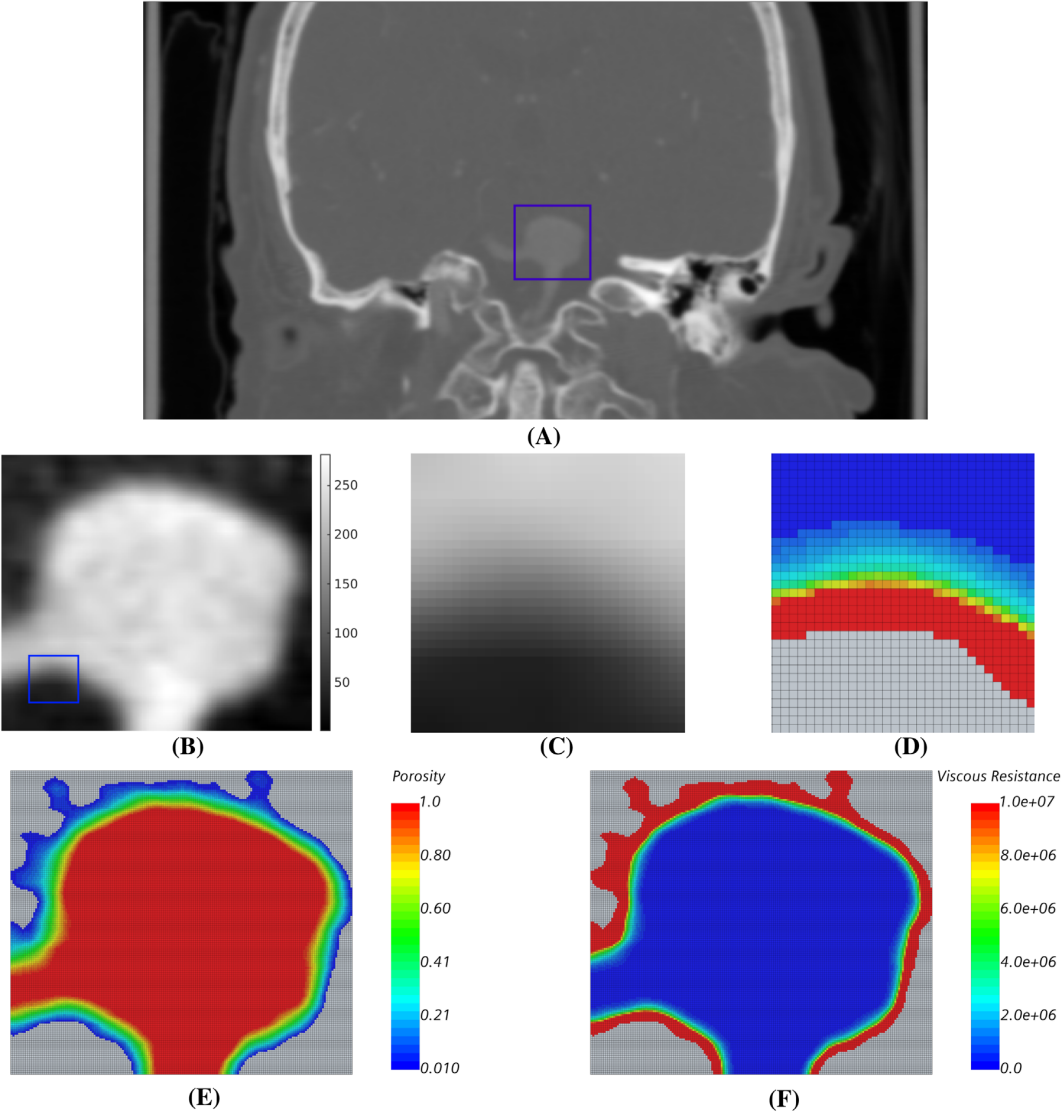
Different stages of the image preprocessing and porous medium setup, for a section of the CA 5 dataset. (A) coronal slice of CT image to identify the aneurysm, (B) cropped and upsampled image, (C) and (D) detailed view of lumen‐tissue interface voxels with intensity and corresponding porous viscous resistance (*kg*/*m* ^3^ *s*), (E) and (F) porosity and porous viscous resistance computed for the cropped image shown in (b)

An example of the results at different stages of the proposed methodology (see Figure 1) are presented in Figure 7, showing a cross‐section of the computed tomography medical images, the model porosity and viscous resistance. The small blue box in Figure 7A identifies a saccular aneurysm as the region of interest, and consequently the image is cropped accordingly. The target aneurysm is magnified in Figure 7B, presenting the image intensity distribution after linear interpolation upsampling. The further small blue box identifies a region of the vessel‐tissue interface which is shown in detail in Figure 7C together with the corresponding modelled viscous resistance in Figure 7D. A smooth transition of viscous resistance from a fully solid to fully fluid region is observed, which depends on the greyscale intensity distribution arising from imaging partial volume effects. The porosity and the resulting viscous resistance for the cropped region are shown in Figure 7E and F. It is evident that there is an inverse relationship between porosity and viscous resistance distribution, given by Equation 63. The transparent (grey) cells seen around the object are voxels considered as entirely solid, and were removed from the computational domain.

**FIGURE 8:**
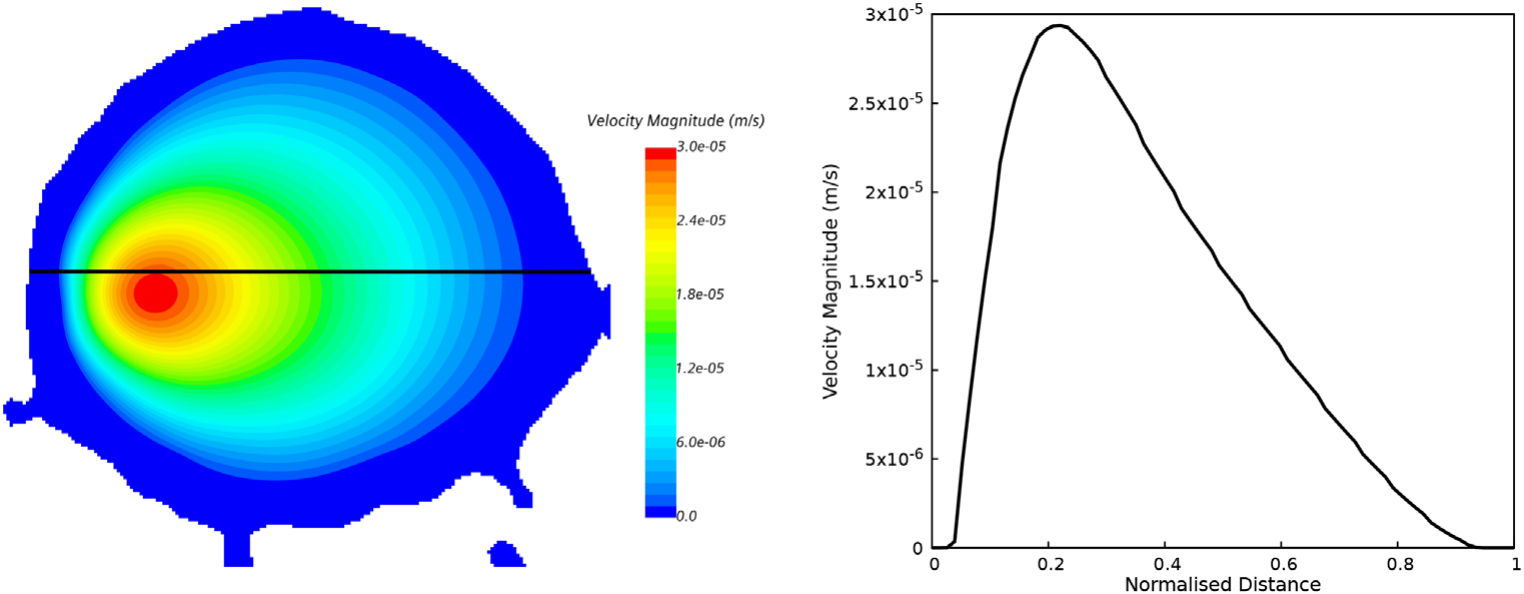
Example CFD result of flow in the model porous medium, here for CA 5 with corresponding section to that shown in Figure 2. Left: map of velocity magnitude. Right: velocity profile along the line indicated

The contour of velocity magnitude and the velocity profile along a line are plotted in Figure 8 (cross‐section locations as in Figure 2 as an example CFD solution for flow in CA 5. Creeping flow is observed in the aneurysm, and the velocity profile shows a gradual and smooth change from the core flow region to the near‐wall region.

### 3.2 Volume change and iso‐suface value

**FIGURE 9:**
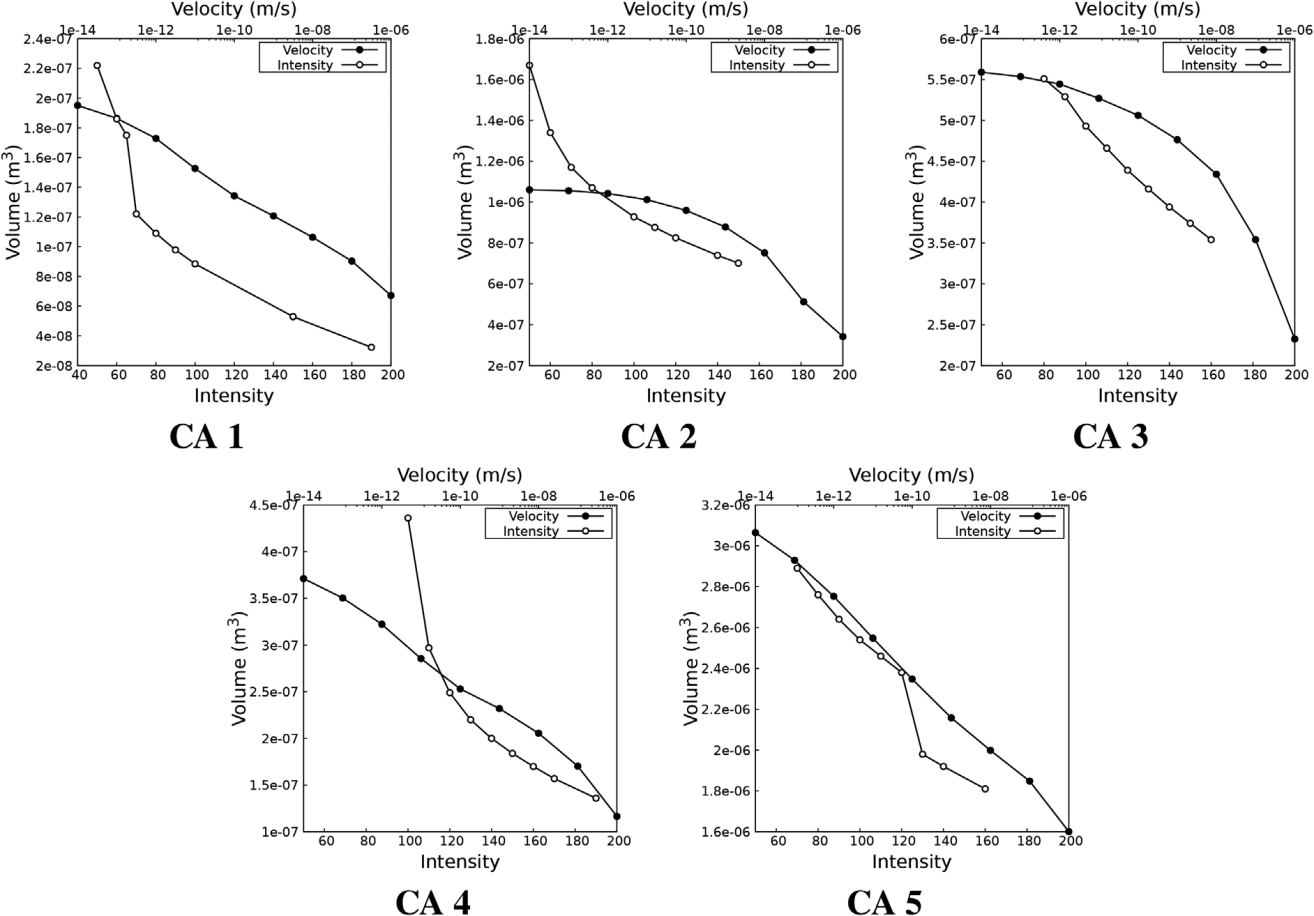
Volume of aneurysm obtained from velocity thresholding and image greyscale thresholding approaches. The top and bottom axes show the velocity iso‐values and minimum limit of segmentations, respectively. See Table 2 for the final threshold values identified

A comparison of the volume of segmented aneurysms obtained from: (i) thresholding a velocity iso‐surface computed from the numerical simulation of the porous medium, and (ii) thresholding a constant image greyscale intensity, for a range of values, are shown in Figure 9. The different rates of volume change with the varying velocity or intensity thresholding value can be clearly appreciated. We also observe that the changes in these curves occur at approximately, though not precisely, at the same values of the segmented object volume irrespective if segmentation is effected by the velocity or intensity thresholding. This result indicates that the object segmentation on the whole is consistently obtained, though the level of noise and its treatment is different, resulting in different object representation upon segmentation.

**FIGURE 10:**
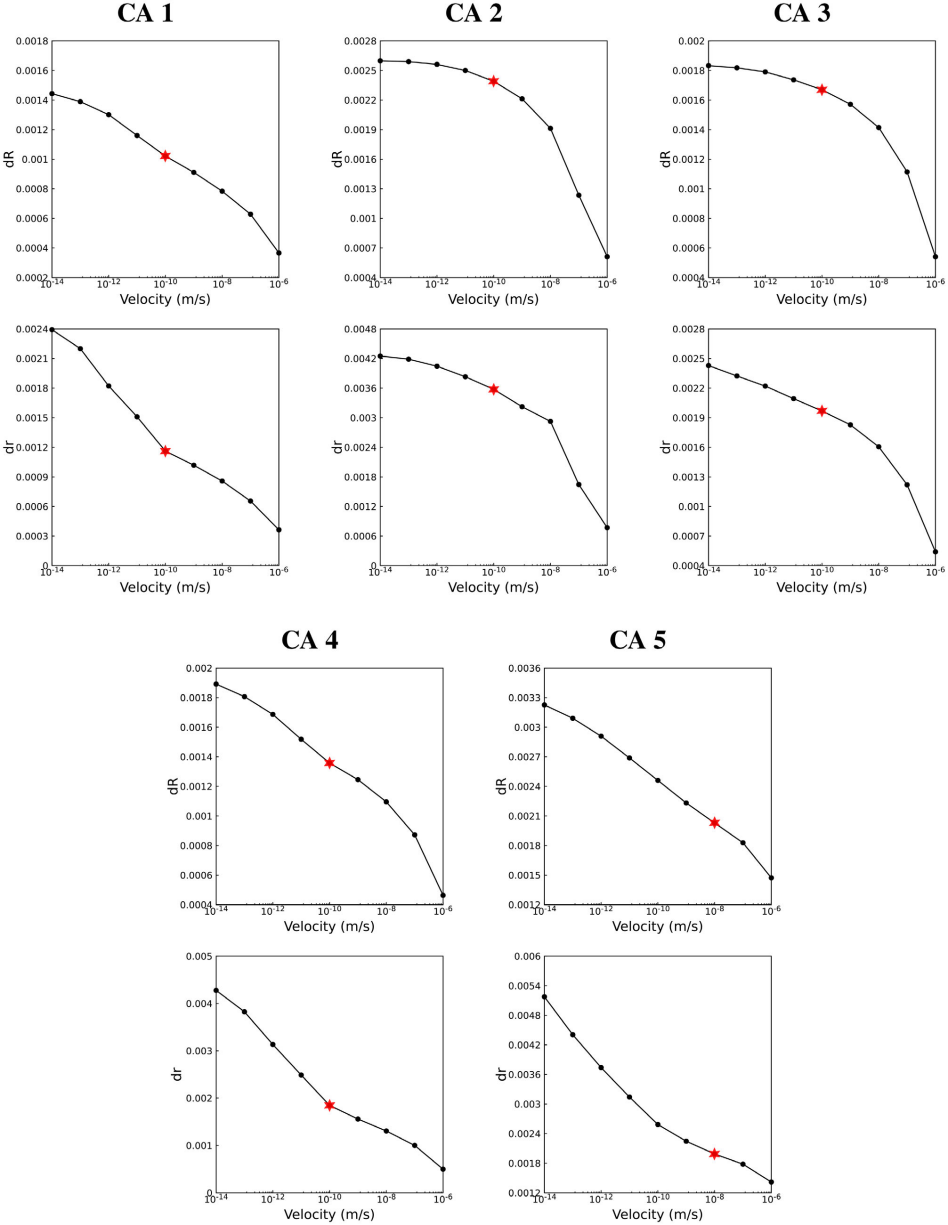
Solution of *dR* and *dr* for CAs 1–5. The initial area and volume were extracted for the iso‐value = 1e‐5 m/s. See Table 2 for the final threshold values identified

The simplified model spherical aneurysm representation, presented in Section 2.5, provides an additional means of identifying a possible range of iso‐values for the segmentation. The roots of Equations 64 and 65 for CAs 1–5 are plotted in Figure 10. We observe identifiable segmentation points in the plots where there is plateau followed by rapid change in the slopes. The plateau regions are inflexion points in these curves, and are indications that the segmented surface definition is on average insensitive to a local variation in threshold value, as is the case when delineating distinct objects with uniform intensities. Consequently these inflexion points identify good candidate velocity thresholding values. In some cases, including CAs 1, 4, and 5, an inflexion in the curves is readily observed, and the values for velocity thresholding are clearly identifiable at this inflexion. In CAs 2 and 3, we do not observe inflexions in the curves, and the reason for this is that the coarse thresholding, as part of the image pre‐processing stage, removed much of the image background and hence no increase in *dR* or *dr* is observed at extreme low velocity thresholding values. In such cases the values for velocity thresholding are clearly identifiable at the start of the plateau region, as if there were an inflexion.

The final selected iso‐surface values for the segmentation are reported in Table 2, and are marked in Figure 10. It is important to note that the iso‐surface value indicator is introduced to guide the appropriate velocity thresholding selection, based on the behaviour we observe in the model spherical object. Therefore, even though it is able to specify a point or range of the iso‐surface values for the segmentation, the result should be verified and adjusted if required. In the present work no adjustment was carried out.

### 3.3 Shape comparison

**FIGURE 11:**
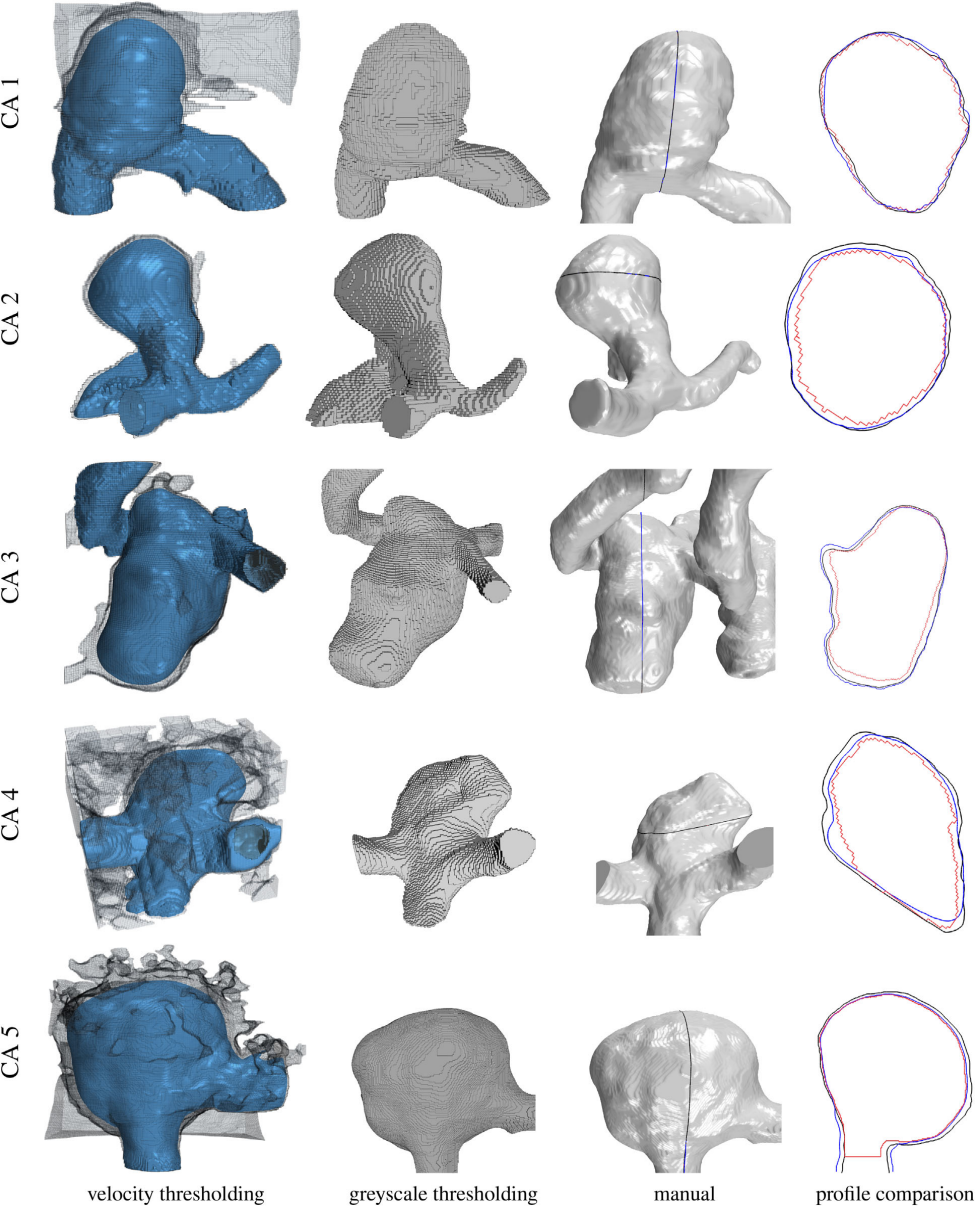
Results of segmentation for CAs 1–5. The first, second, and third column is the outcome of velocity thresholding, image greyscale thresholding, and manual segmentation. The iso‐values used in velocity thresholding and the threshold limits are presented in Table 2. The fourth column is the profile comparison at an arbitrary section: black‐manual segmentation; blue‐velocity thresholding segmentation; red‐constant threshold segmentation

The results of velocity thresholding, image greyscale thresholding and manual segmentation, along with cross‐section comparisons, are shown in Figure 11. The first column in this figure presents the velocity iso‐surfaces embedded in the computational domains, which are transparent (grey) in the figure. From both visual inspection and comparison of cross‐sections, we observe that the velocity thresholding method is effective at extracting the aneurysm surfaces and provides a close comparison to the manual segmentation results. The surface of the aneurysms are smooth and the arteries preserve more details, which is not the case for the intensity thresholding.

A comparison of the segmented surfaces can be effectively carried out by measuring the *cloud‐to‐mesh* (C2M) distance, taking the manual segmentation as reference. C2M results are computed by CloudCompare Open‐Source Software (version 2.11.1).^58^ Table 3 presents the normalised (based on pixel size) mean, maximum, and standard deviation of C2M distance. The mean and standard deviation C2M results are less than one for all the aneurysm datasets investigated, hence below the voxel resolution. However, local regions where C2M distance is greater than one may be present, though these are focal spots and may be related to imprecise manual segmentation. A comparison with widely used segmentation approaches such as active contour, fast marching, Canny edge detection, watershed, region growing and the proposed method is provided in the Appendix.

**TABLE 3:** Statistical shape comparison of extracted aneurysms from velocity thresholding against the manual segmentation results. The values are normalised based on pixel size (hence the scanning section resolution) reported in Table 1

	Mean distance	Max distance	Standard deviation
CA 1	0.03	1.28	0.29
CA 2	0.33	1.41	0.25
CA 3	0.02	1.84	0.37
CA 4	0.15	2.15	0.39
CA 5	0.41	1.63	0.59

### 3.4 Sensitivity analysis

**FIGURE 12:**
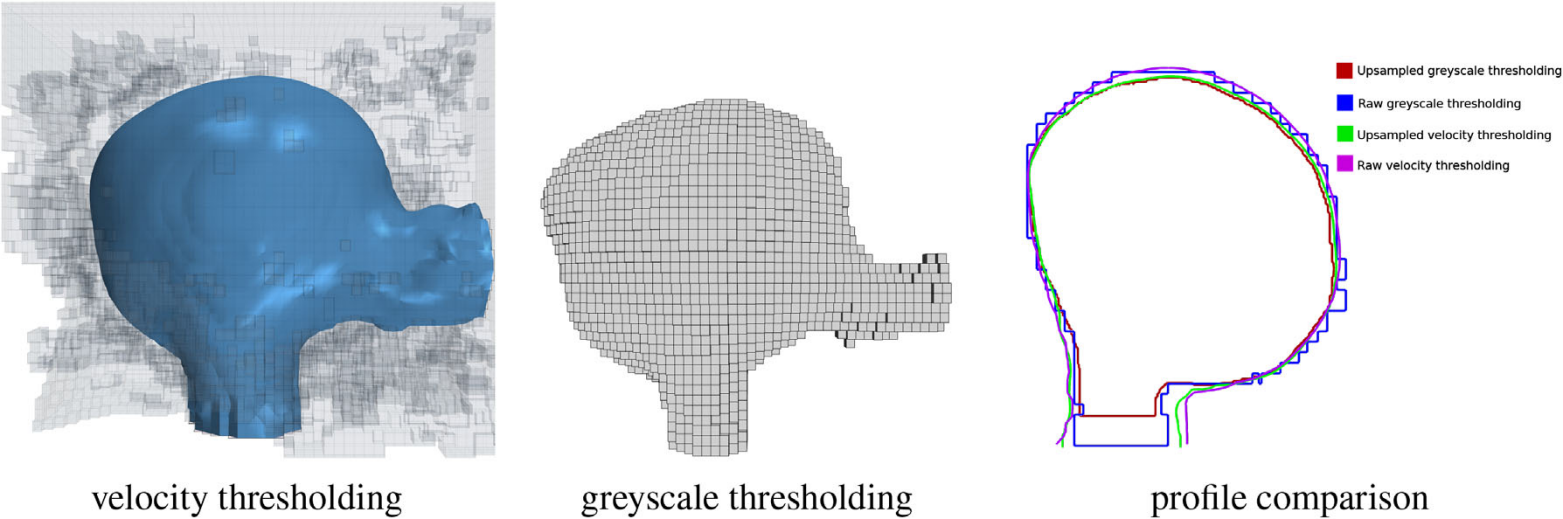
Results of segmentation for the original image (no upsampling) of CA 5. The cross‐section profiles obtained from velocity thresholding and greyscale thresholding are also compared. The results with upsampling and the location of the cross‐section is shown in Figure 11

The proposed velocity thresholding has been tested on a range of medical image datasets, however some algorithmic steps and user‐defined parameters may lead to alternative segmentations. A sensitivity study is therefore necessary, and this may provide further insight on the workings of the method. Firstly, the method was tested on the original image (no upsampling) of CA 5 as shown in Figure 12. While the velocity segmentation for the original and upsampled domains compare well, we observe some details present in the upsampled segmentation are missing or appear accentuated in the original image segmentation. The reason for this is that the coarser resolution is unable to resolve features fully, and approximates them in this fashion.

**FIGURE 13:**
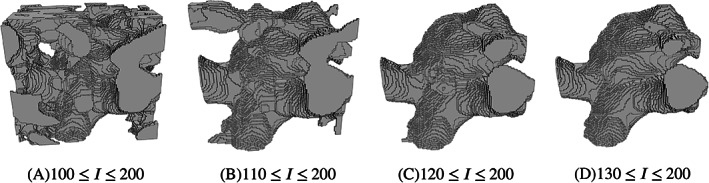
Greyscale thresholding segmentation for CA 4. Various minimum limits from *T* = 100–130 are selected

As part of the image pre‐processing stage, after the user identifies the region of interest, a coarse intensity thresholding is performed. This thresholding reduces the computational cost and importantly sets the range for the translation from intensity to porosity. Since this is a user parameter it requires further investigation and explanation. Example segmentations obtained from simple image greyscale thresholding for CA 4 is presented in Figure 13. We observe that the lower threshold value of intensity *I* = 100 is prone to noise artefacts, while the value of intensity *I* = 200 is identified as the largest value in the region of interest. One can then conclude that the true aneurysm boundary for this image dataset is a subset of the voxels in the range 100 ≤ *I* ≤ 200. We observe from Figure 13 that the quality of segmented geometry, in terms of noise exclusion, significantly depends on the minimum limit of intensity. The coarse intensity thresholding for CA 4 was chosen as 100 ≤ *I* ≤ 200 (see Table 2, hence with a comfortable margin to ensure the object lies within intensity range. We can conclude that a finer resolution is therefore advisable, though the final segmented object surface will nonetheless be very good even at the lower resolution.

**FIGURE 14:**
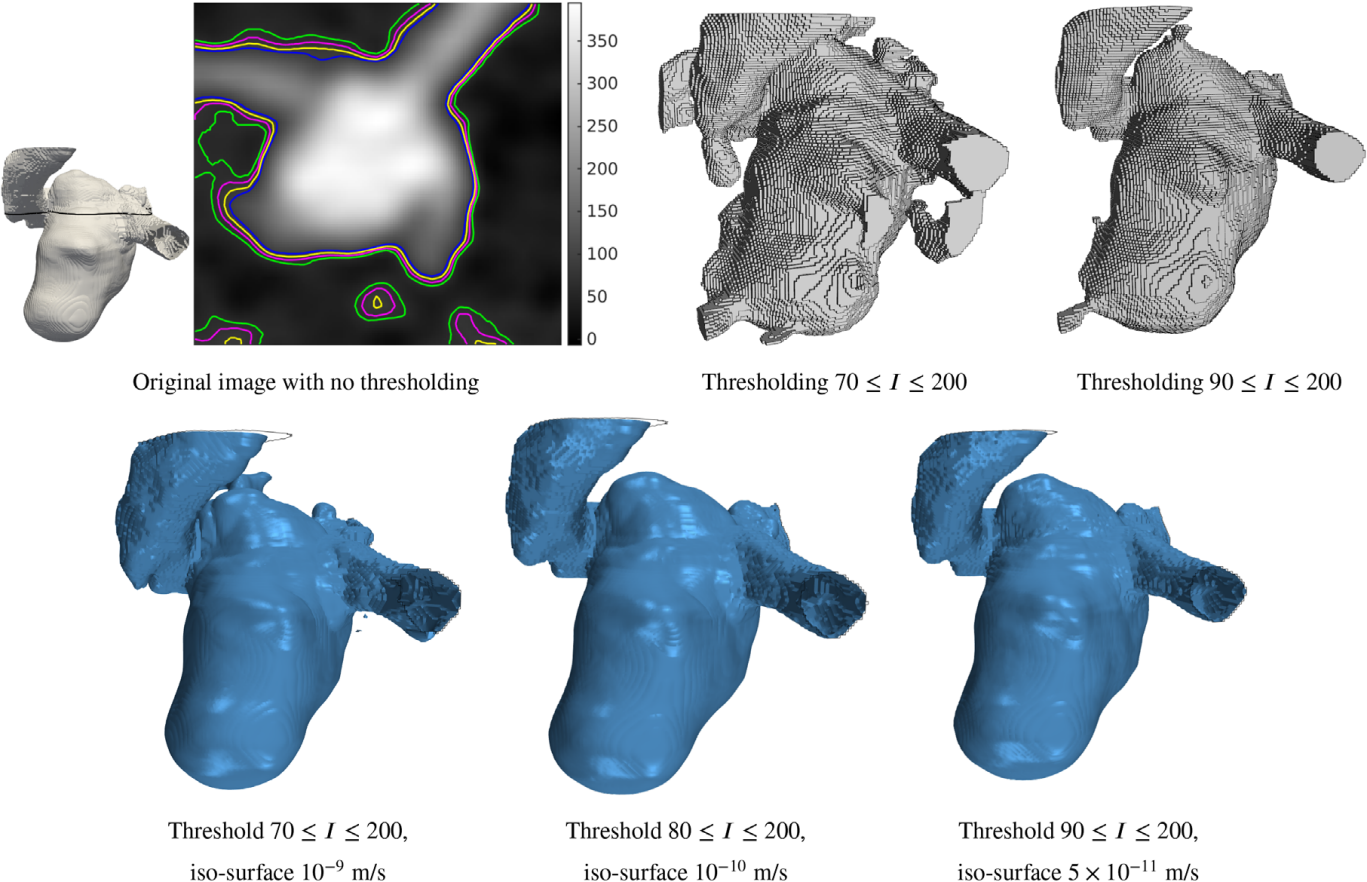
Sensitivity analysis of CA 3. (A) slice of the image with contours of aneurysms boundary for different minimum limits: blue, yellow, pink, and green lines represent the profiles for 90, 80,70, and 60 as minimum limits, respectively. The location of the cross‐section is also presented. (B) and (C) are greyscale thresholding segmentation. (D), (E), and (F) are the result of velocity thresholding segmentation

A sensitivity analysis of the coarse thresholding interval selection during the image preprocessing stage is detailed for CA 3, in which lower thresholding values of *I* = 70, 80 and 90 are considered. Figure 14A compares the cross‐section profiles of the aneurysm for these different values and the surface of the aneurysms are shown in Figure 14B, C. The results of velocity iso‐surfaces segmentations for these computational domains are presented in Figure 14D–14F, from which we note that there are negligible differences in the segmentations. An additional surface detail is observed in the arteries when the computational domain is defined by the *I* = 70 minimum limit, which is due to the presence of more voxels in the computational domain. The identified velocity iso‐surface value starts from 10^−9^ m/s for the *I* = 70 bound, 10^−10^ m/s for *I* = 80 bound, and 5 × 10^−11^ m/s for *I* = 90 bound. This gradual increase again arises from Equation 4 that computes porosity from image intensity. Reducing the minimum limit introduces more resistance and undesired voxels to the domain, which is relative to minimum and maximum of intensity. We can conclude that the user choice of coarse intensity thresholding is insensitive to the final segmented object surface.

A sensitivity study investigating the working fluid properties and subsequent velocity segmentation results was also carried out. The strength of viscous and inertial losses in a porous medium can be quantified by the Blake number, which is a modified Reynolds number for a porous materials and is defined by

(66) $$\mathrm{Bl}=\frac{\rho {\mathrm{uD}}_{h}}{\mu \left(1-\varepsilon \right)}$$

Using the hydraulic radius for a shrunken voxel (Equation 50), we get

(67) $$\mathrm{Bl}=\frac{\mathrm{\rho ud}}{\mu }\frac{\varepsilon }{\left(1-\varepsilon \right)}$$

**FIGURE 15:**
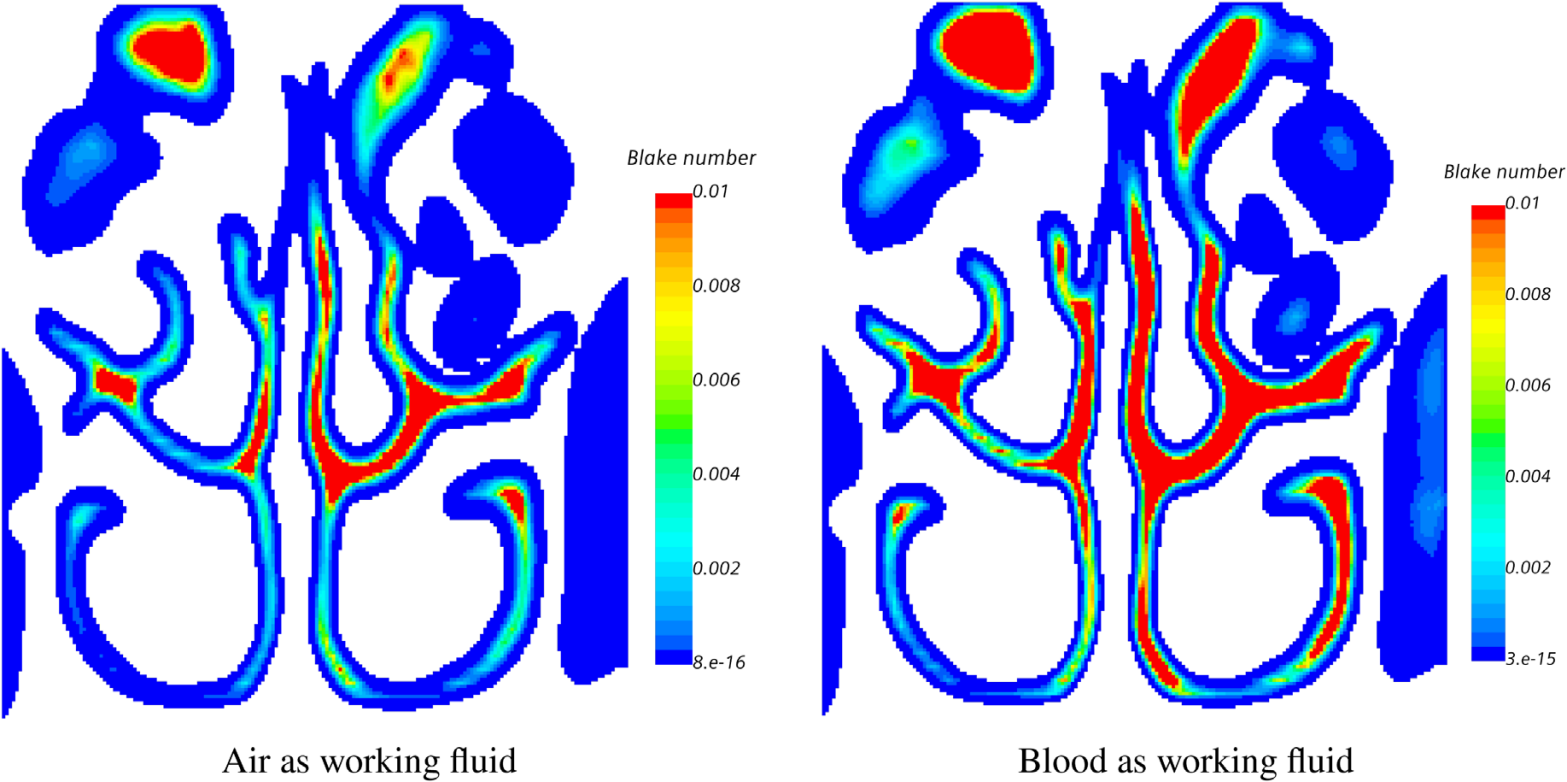
Map of Blake number on cross‐section *B* for NA 1 for different working fluids (*air* and *blood*). The location of cross‐section *B* is shown in Figure 16

**FIGURE 16:**
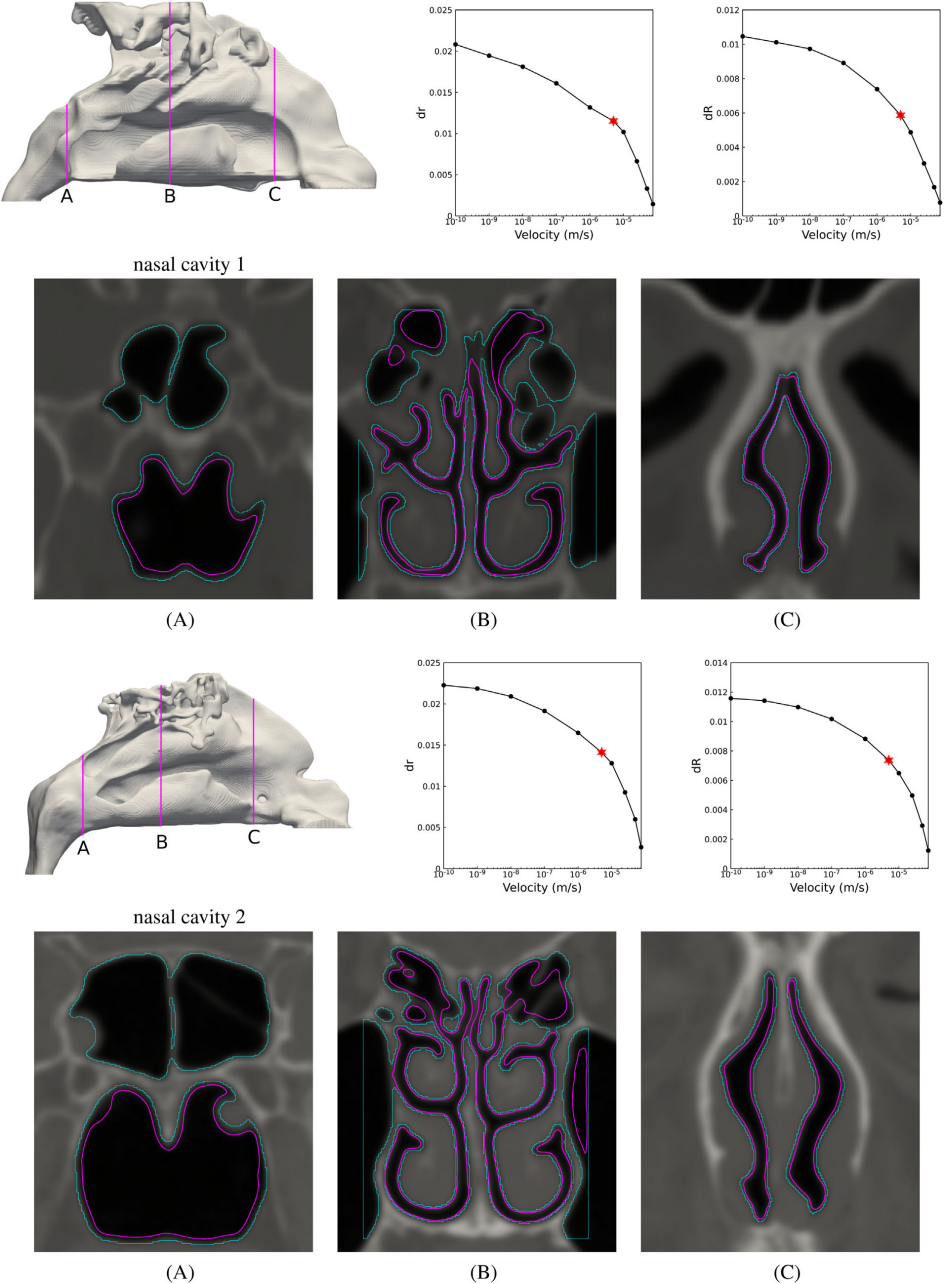
Result of velocity thresholding segmentation of two patient‐specific nasal cavity anatomies. Top row: the velocity iso‐surface of |**u**| = 5 × 10^−6^ m/s and the solution of *dR* and *dr*, respectively. The initial area and volume were extracted for the iso‐value |**u**| = 10^−4^ m/s. Bottom row: Segmentation of the image on three different cross‐sections where light blue is the border of the computational domain and pink is the profile of the iso‐surface considered as the result of the segmentation. The location of the cross‐sections is presented

Each voxel, considered as a channel, has its porosity and fluid velocity. As such, the Blake number can be computed for each voxel, and in Figure 15 the Blake number for NC 1 and two working fluids, including *air* and *blood* (*ρ*_*blood*_ = 1030 kg m^−3^, *ρ*_*air*_ = 1.18415 kg m^−3^, *μ*_*blood*_ = 0.004 N s m^−2^, *μ*_*air*_ = 1.85508*e* − 5 N s m^−2^), is plotted on cross‐section B (see Figure 16) as an example. We observe that for both working fluids the Blake number is of the order of *Bl* = 10^−7^ for near‐wall voxels, indicating that the viscous resistance is dominant in this region. Therefore, the segmentation outcomes are not sensitive to the working fluid properties. Consequently the working fluid is used merely to fill the domain, obtain sub‐voxel resolution and extract the region of interest by velocity iso‐surface thresholding. As such, the proposed approach can also be employed for other objects of interest, irrespective if a fluid‐tissue interface would exist in reality. We can conclude that the final segmented object is not sensitive to the fluid properties in the porous medium, as long as the Reynolds number remains low to avoid inertial effects.

### 3.5 Model performance on more challenging datasets

**FIGURE 17:**
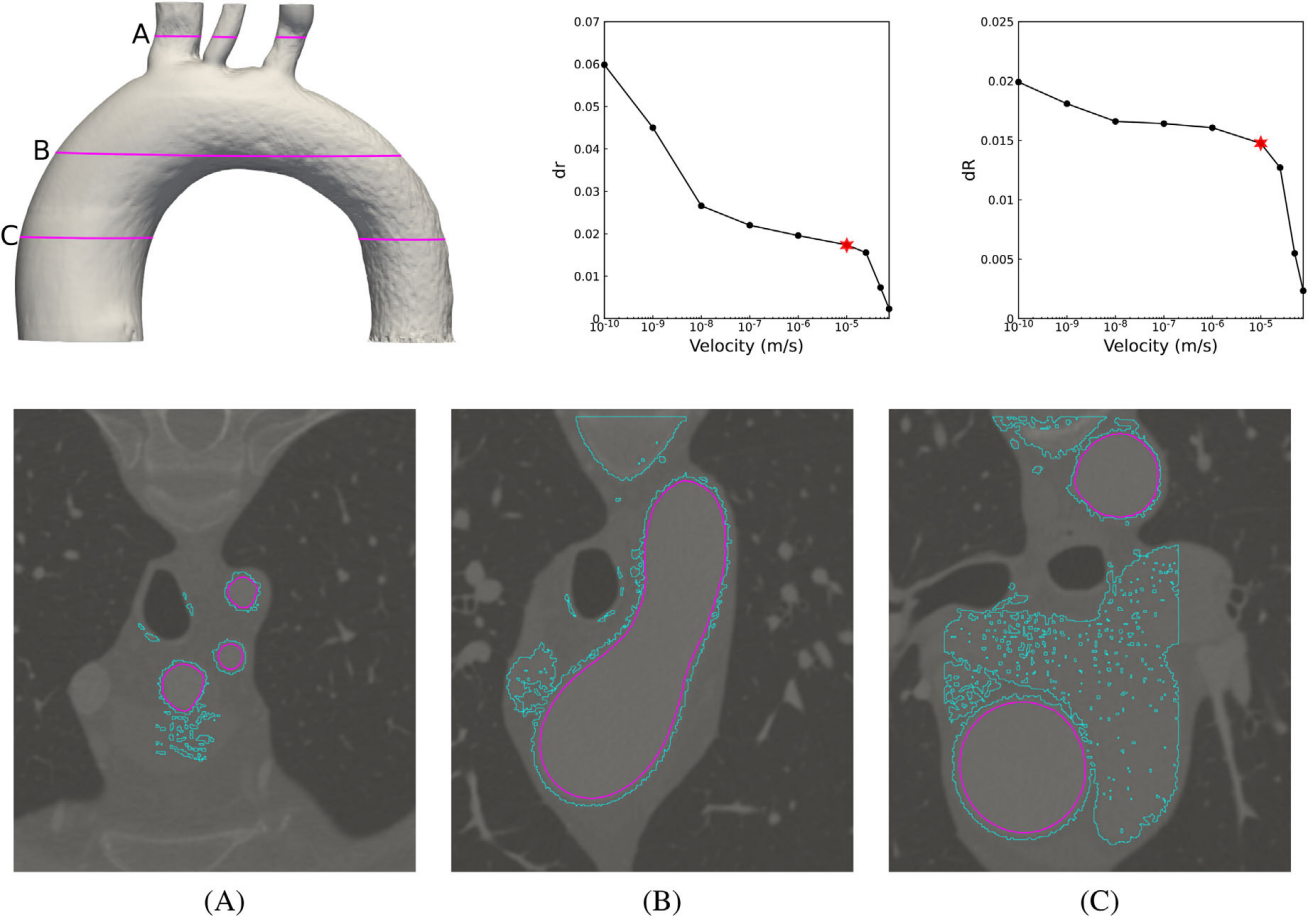
Result of velocity thresholding segmentation of aorta. Top row: the velocity iso‐surface of |**u**| = 10^−5^ m/s and the solution of *dR* and *dr*, respectively. The initial area and volume were extracted for the iso‐value |**u**| = 10^−4^ m/s. Bottom row: Segmentation of the image on three different cross‐sections where light blue is the border of the computational domain and pink is the profile of the iso‐surface considered as the result of the segmentation. The location of the cross‐sections is presented

Having evaluated in detail the performance of the proposed method for the CA datasets, we turn our attention to segmenting other more complex datasets. Specifically, we consider CT images corresponding to two NC and one AA. The image dimensions are reported in Table 1. These anatomies are of interest here particularly since they exhibit a high degree of morphological variability. Some regions, such as meatuses and septal passage in the upper airways require fine spatial resolution for numerical simulations, making it necessary to upsample the image to have several computational cells across each passage. As such, in the NC cases, the voxel size was refined to have (0.25)^3^ mm, resulting in approximately 4 and 4.5 million cells, for NC 1 and NC 2 respectively. The AA case has wider passages when compared to the nasal cavity, making it possible to perform the segmentation without upsampling. As a result the AA 1 case has approximately 2 million computational cells. The inlet velocity was set to |**u**| = 10^−4^ m/s, and the Reynolds number and threshold limits are again reported in Table 2. The outcome of the velocity segmentation, the solution of *dr* and *dR* and three cross‐sections through each test case, are presented in Figures 16, 17.

We observe that the method properly extracts the shape of nasal cavity and preserves important features such as meatuses, even in the presence of connections with surrounding cavities in the computational domain. This is where the porous viscous resistance effectively comes into play. However, the method struggles to separate upper parts of the septal passage in NC 1 (see cross‐sections B and C in Figure 16), which is due to narrow anatomy (hence inherently slow flow) and evidence of a physical obstruction. In such drastic cases, the velocity segmentation needs to be subsequently adjusted. The solution of *dr* and *dR* suggest |**u**| = 5 × 10^−6^ m/s as target iso‐surface value for the segmentation, with *dr* as a slightly better indicator for detecting the segmentation value. The reason is that in such complex and scroll‐like geometries the object area is more sensitive to the thresholding values compared to the volume.

In the AA case, we clearly observe the excessive presence of noise and unwanted voxels in the computational domain, however the velocity threshold segmentation works well and the shape of the aorta is successfully extracted. Importantly, the method is seen to handle branching objects in a straightforward manner. The velocity iso‐surface value is identified from the plots of *dr* and *dR* clearly as |**u**| = 10^−8^ m/s, which appears reasonable from visual inspection.

### 3.6 Computational cost

The computational cost is an important aspect of any segmentation approach. The steps of cropping and thresholding, discussed in section 2.1, aim to address this concern by reducing the number of numerical cells and thereby reducing the computational cost. Also, as mentioned above, the flow is considered to be steady‐state and is in the creeping regime, helping to maintain simplicity and effectiveness. Table 4 shows the convergence time for series and parallel simulations. The parallel processing significantly reduces the run time. The required time for the solver to obtain proper velocity iso‐surface varies for anatomies with different levels of morphological complexity. In fact, the computational cost not only depends on the number of computational cells but is also related to the geometry in which the working fluid flows. This arises from the physical nature of the approach. A general purpose Finite Volume solver may not be the best suited for such grid configuration in terms of computational efficiency, and we note that specialised grid‐based methods such as the LBM or finite difference method (FDM) typically boast higher computational efficiency.

**TABLE 4:** Number of computational cells and the run time (wall time) for the serial and parallel simulations. Parallel processing was performed using 16 Intel Xeon 2.1 GHz (E5‐2620 v4) processors

	Computational cells (million)	Run time (min)	
		Serial	Parallel
CA 1	0.2	3.5	1
CA 2	1.1	10	2
CA 3	0.53	8	1.5
CA 4	0.44	2.5	0.8
CA 5	3.4	15	5
NC 1	4.5	20	4
NC 2	4	18	3
AA 1	2	5	1

### 3.7 CFD application: A case study

**FIGURE 18:**
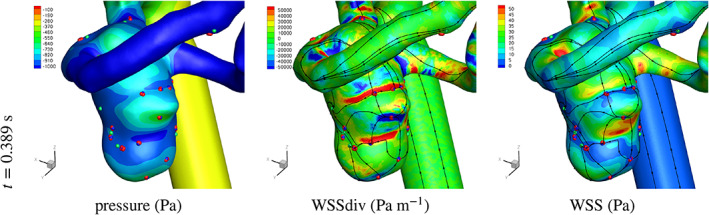
Solution of pressure, WSSdiv and WSS for CA 3. Plots of surface shear lines and WSS critical points. The results are presented and compared with the previous work reported in^35^

Having obtained encouraging segmentation results, both visually and by comparison to manual segmentation, it is worth investigating the subsequent use of the virtual models. As an example, we undertake computational haemodynamic simulations selecting CA 3 for this test as it has one of the most complex shapes among the CA datasets. We adopted the technique introduced in^35^ to study the flow in aneurysms and follow the setup detailed therein. The output of the segmentation was smoothed using the bi‐Laplacian method in order to remove the small irregularities arising from the marching cubes iso‐surface tessellation and the linear upsampling of the medical image,^2^, ^59^ and the inlets and the outlet were extruded in the coaxial direction. In previous work^35^ we analysed near‐wall flow structure and wall shear stress critical points for the same CA datasets and in Figure 18 a time snapshot of these results is presented. The near‐wall fluid mechanics measures are especially sensitive to the morphology of the no‐slip boundary, and we encouragingly find good agreement with the results discussed in,^35^ indicating that the proposed velocity thresholding segmentation is sufficiently accurate and reproducible for these studies.

## 4 CONCLUSION

A novel, physics‐based approach for medical image segmentation is presented, which is overall robust and can be readily generalised to various segmentation tasks. The method works by translating the medical image dataset to a computational domain, where the greyscale intensity at each voxel is interpreted as a porous resistance. The resistance is based on the shrunken voxel model which depends on constants *A* and *B*, and these are respectively given unambiguously by Equations 4 and 63. Indeed the value of these constants depends solely on the value of porosity, which in turn is not a free parameter for the user to set. The user inputs are solely the choice of the lower and upper bounds of intensity for the object of interest, which may be obtained in a straightforward fashion (or indeed automatically) after cropping the image to isolate the region of interest. CFD simulations through this porous medium provides a velocity field, and a velocity magnitude iso‐surface then provides the object surface definition. We obtain sub‐voxel resolution on segmentation, since the fluid velocity distribution in a porous medium varies smoothly, and the surface definition obtained is of high quality.

The method was first verified on five patient‐specific CA, and the results were compared with both manual (gold standard) and greyscale thresholding segmentation. A sensitivity analysis on all user‐defined choices with respect to the segmentation quality indicated the method is robust and repeatable. Results of a computational haemodynamics simulation on a segmented cerebral aneurysm geometry was compared to previous work, and indicated faithful comparison to the near‐wall fluid mechanics parameters which are known to be sensitive to the surface definition. Consequently, the results of the segmentation are suitable for use in numerical simulations and other post‐segmentation evaluation.

The method was then used to segment two NC and one AA as more challenging objects of interest, with no change to the methodology and hence a generalisation of the target objects. Encouraging results were obtained, showing some limitations when medical image resolution is locally extremely poor, but otherwise the method is resilient to the presence of noise. It is worth noting that the developed approach is not only applicable for segmenting regions of the cardiovascular or respiratory systems. Indeed the porous model can be adopted for segmenting other objects of interest, hence further generalisations are possible.

The computational cost is modest, since steady‐state CFD solutions for flow in the porous medium is required. We envisage that employing solvers well suited to such grid‐based discretisations, such as the LBM or FDM, together with parallelisation using GPGPUs, the method will be practical and versatile.

Finally, we note that the conceptual working on the method holds parallels with the VoF or immersed boundary methods, and instead of a porous medium one could consider a secondary fluid to regulate the viscous resistance, such that fluid properties in each voxel are calculated by a volume fraction average of all fluids. While the authors have not investigated this further, we foresee similar successful segmentation results once the voxel partial volume considerations are correctly translated to the computational models. Additionally, it would be interesting to explore alternative wall boundary conditions for the CFD simulations of flow in the porous model, namely a slip‐flow tangent to the medical image intensity iso‐surface. This could be achieved by adopting a Beavers‐Joseph interface condition and would avoid development of the Brinkman layer,^60^ consequently facilitating the choice of the velocity magnitude for iso‐surface segmentation.

## Figures and Tables

**FIGURE A1:**
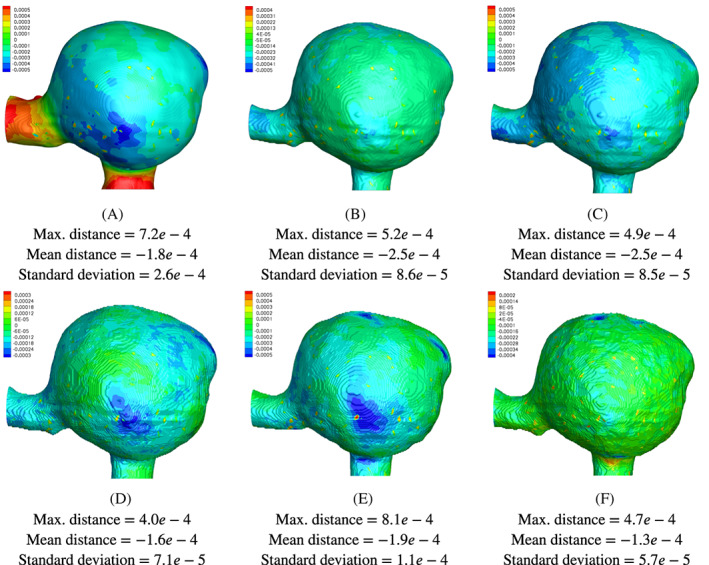
Contour of C2M distance with manual segmentation as reference for (a) velocity thresholding model, (b) active contour (snakes), (c) Canny edge detection, (d) region growing, (e) watershed, and (f) fast marching methods. Maximum distance, mean distance, and standard deviation is reported

**FIGURE A2:**
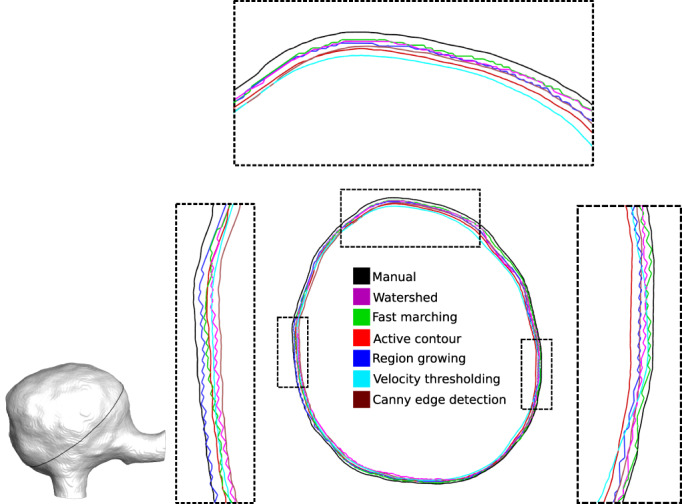
Cross‐section profile comparison of CA 5 for the velocity thresholding model, active contour (snakes), Canny edge detection, region growing, watershed, and fast marching methods. The location of the cross‐section is presented

## Data Availability

The data that support the findings of this study are available on request from the corresponding author. The data are not publicly available due to privacy or ethical restrictions.

## APPENDIX A

### A.1 A Methods comparison

In this section, a comparison is made between the proposed method and other well‐developed techniques, including active contour, fast marching, Canny edge detection, watershed, and region growing. The segmentations were carried out using 3D‐Slicer^22^ and ITK‐SNAP,^19^ and considerable care with trial and error adjustments to parameters were required to provide the best possible segmentation with these well‐developed methods. Dataset CA 5 was chosen for the comparison due to its robustness compared to more challenging ones like CA 3, for which some of the methods listed above failed to produce reasonable results. No post‐processing was performed after the segmentations in order to provide a fair and direct comparison. The *cloud‐to‐mesh* (C2M) distance computed by CloudCompare Open‐Source Software (version 2.11.1)^58^ was used as the comparison metric for three‐dimensional segmented shapes. Contours of C2M distance are presented in Figure A1 where the manual segmentation is considered as the reference (gold standard). The maximum, mean and standard deviation of C2M distance are reported in Figure A2 together with the cross‐section profile comparison.

As observed, the results are satisfactory for all methods tested, and are in good agreement with the reference surface. The outcome of velocity thresholding has the smoothest surface morphology among the shapes, without resorting to further post‐processing. However, we observe that the parent arteries are wider, which is caused here by the proximity of the inlet and outlet boundaries to the aneurysm (see Figure 6).

This comparison shows the capability of the proposed method in medical image segmentation. It ensures reasonable segmentation even for the most drastic cases without the need for careful parameter selection. Indeed the method appears robust in the numerical tests carried out. It is a physical‐based technique, which may be readily developed further and relies on little user intervention.
